# Treatment of non-small cell lung cancer using chem-bioinformatics-driven engineering of exosomal cargo-vehicle for telmisartan and pioglitazone targeted-delivery

**DOI:** 10.1038/s41598-025-10416-0

**Published:** 2025-07-11

**Authors:** Nadia M. Hamdy, Eman F. Sanad, Shaymaa E. Kassab, Merhan Essam, Monica A. Guirguis, Emad B. Basalious, Ahmed S. Sultan

**Affiliations:** 1 Department of Biochemistry, Faculty of Pharmacy, Ain Shams University, Abassia, 11566 Cairo Egypt; 2 Department of Pharmaceutical Chemistry, Faculty of Pharmacy, Damanhour University, Damanhour, 22516 Egypt; 3 Protac Scientific, Drug Discovery Pro LLC, Cairo, 11627 Egypt; 4 Biochemistry Department, Faculty of Science, Alexandria University, El- Shatbi, 21568 Alexandria Egypt; 5 Department of Oncology, Lombardi Comprehensive Cancer Center, Georgetown University Medical Center, Washington, DC USA; 6 Department of Pharmaceutics and Industrial Pharmacy, Faculty of Pharmacy, Cairo University, Al Kasr AlAiny, Cairo, 11562 Egypt

**Keywords:** Bioinformatics, Cheminformatics, Pioglitazone, Telmisartan, Lung adenocarcinoma, Exosomes, PPARG, Targeted delivery, Molecular Docking, In Silico drug repurposing, Biochemistry, Biotechnology, Cancer, Computational biology and bioinformatics, Drug discovery, Genetics, Immunology, Molecular biology, Biomarkers, Diseases, Medical research, Molecular medicine, Oncology, Pathogenesis

## Abstract

The activation of the PPARG transcription factor is linked to reduced non-small cell lung cancer (NSCLC) growth. Bioinformatics, cheminformatics, and molecular docking/dynamics studies assessing pioglitazone and telmisartan as repurposed PPARG agonists for treating NSCLC with a targeted delivery system was done. Bioinformatics confirmed that the expression of the PPARG gene can predict outcomes in lung adenocarcinoma and is related to immune cells present in the tumor. Cheminformatics data showed that pioglitazone and telmisartan have a strong attraction to the PPARG receptor, with good efficiency as ligands. Both drugs were found to be lipophilic, suggesting compatibility with a targeted delivery formulation that may include albumin. Further cheminformatics predictions highlighted systemic toxicity values and the need for targeted delivery to minimize toxic side effects. Molecular docking and dynamics simulations showed that the telmisartan-MyoVc cargo domain complex was strong and stable during an 18 ns simulation period. Bioinformatics and cheminformatics data support pioglitazone and telmisartan as promising repurposed drugs for LUAC, highlighting their lipophilicity and compatibility with exosomal components like albumin. Cheminformatics also pointed out potential off-target effects and hepatotoxicity, emphasizing the importance of exosomal targeted delivery. Molecular docking and MD simulations confirmed the affinity and stability of drug-exosomal vehicle complexes. The proposed engineering of exosomal cargo for targeted delivery of these drugs to lung cells could enhance NSCLC treatment and address drug resistance while minimizing systemic toxicity.

## Introduction

### Problem statement

The creation of an effective system/formula for treating lung cancer is critical, especially given the annual increase in new cancer cases.

### Literature review

Cancer-related fatality impedes progress toward Sustainable Development Goal (SDG) #3 of “Better Health.”

As the field of drug delivery systems (DDS) has evolved, nanotechnology (NT) has made significant contributions to the development of smart nanocarriers^1,2^ such as synthetic lipid-based nanocarriers that provide a unique platform for drug encapsulation^3,4^. Furthermore, natural cell-derived carrier systems have sparked substantial interest^5^.

**Fig. 1:**
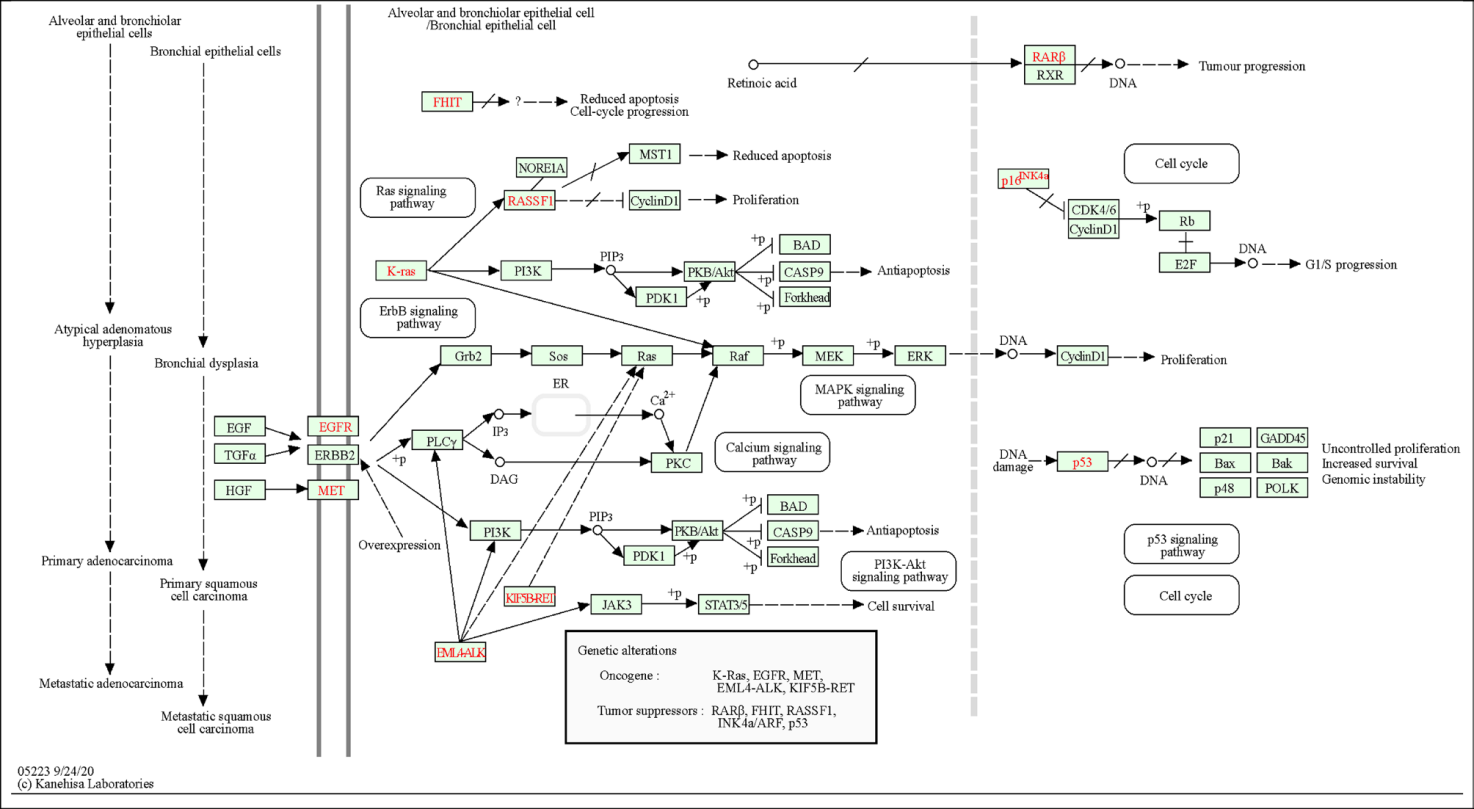
Lung genetic alterations and related pathways retrieved from KEGG, where Ras or ErbB, MAPK, calcium, and PI3K-AKT signaling pathways comprising either oncogenic or tumor suppressor genetic alteration signaling pathways give rise to atypical hyperplasia, then primary and metastatic adenocarcinoma.

The lncRNADisease database^6^ and the Kyoto Encyclopedia of Genes and Genomes (KEGG) database^7^ include these types of lung cancer: lung adenocarcinoma (LUAD), lung cancer that has spread to the brain, lung squamous cell carcinoma, pulmonary adenocarcinoma, non-small cell lung cancer (NSCLC), and small cell lung cancer (SCLC). Genetic changes in the lung and related pathways **(**Fig. 1**)** from KEGG include Ras or ErbB, MAPK (mitogen-activated protein kinase), calcium, and PI3K-AKT signaling pathways, which are linked to oncogenes or tumor suppressor genes.

Interestingly, both in vitro^8–10^ and in vivo^11^ studies have shown that activating peroxisome proliferator-activated receptor gamma (PPARG) slows down the growth of NSCLC lung cancer cells and/or might help stop the spread of developed NSCLC^12^. KEGG data on lung cancer genetic pathways that matched the results of earlier experimental studies, which demonstrated MAPK’s regulatory capability over PPARG activity^13, 14‚15^ providing proof-of-concept for the direction of our research.

Repositioning diverse pharmaceuticals with distinct modes of action is a strategic approach to mitigate or postpone cancer treatment resistance^15^. Selective PPARG activators have been previously investigated for their roles as antidiabetic agents or insulin sensitizers and their involvement in lipid metabolism^16,17^. The efficacy of thiazolidinediones (TZDs), a category of oral PPARG activators that mitigate tumor growth progression in the liver^18^ and in NSCLC xenograft animal models^11,19,20^, has been established for decades. BioGRID^4.4^^21^ and the integrated repository portal for tumor-immune system interactions (TISIDB)^22^ consistently identified pioglitazone as a distinctive interactor of PPARG, classified as a member of TZDs with the most extensive safety margin. Telmisartan, an angiotensin II receptor type 1 (AT1) antagonist, utilized in hypertension management, was identified as a partial PPARG agonist^23–25^. Moreover, recent studies have demonstrated the anticancer properties of telmisartan against many cancer types, including NSCLC^26–28^. Unexpectedly, the PI3K/AKT signaling pathway^28^ contributed to the anticancer efficacy of telmisartan against NSCLC with PPARG activation, aligning with the lung oncogenes depicted in Fig. 1.

The use of exosomes, as a regenerative-biological cell therapy, shows promise for treatment of various non-communicable diseases (NCDs)^29^. We will use exosomes to transport natural or manufactured anticancer drugs, to tumor cells as safe and non-immunogenic way^30–32^. It would be more appropriate to utilize exosomes, which are naturally occurring extracellular vesicles (ECVs) in the blood or tissues, instead of drug-encapsulated nano-scaffolds made of blended FDA-approved polymers like polyethylene glycol (PEG) or polylactic-co-glycolic acid (PLGA).

Online databases Vesiclepedia and Exocarta^33^ show that exosomes, which are a type of extracellular vesicle (ECV), contain parts of cell membranes, such as transmembrane proteins, surface markers, albumin, growth factors and their receptors, proteoglycan receptors, cytokines, heterotrimeric G proteins, heat shock protein 90 (HSP90), and non-muscle myosin^34^. This non-muscle myosin probably works with cytoskeletal actin in target organs, along with adhesion molecules and proteins from the extracellular matrix.

### Aim and objectives

Per, we emphasize pioglitazone and telmisartan as distinctive ligands for the NSCLC repressor PPARG in the current in-silico targeted delivery investigation; therefore, we will use chem-bio-informatics^35^ to find out how well the chosen drugs work for treating lung cancer when delivered by exosome cargo, before testing them in the lab.

Second, to establish the drug’s affinity for the carrier protein, we will run the test drugs through a stiff molecular docking process in the exosome cargo domain, using albumin as a protein component of the target exosome. In addition, we will run a molecular dynamics simulation on the drug-cargo combination to see how stable it is when interacting with the protein in the drug delivery system.

## Materials and methods

### Bioinformatics databases

#### Biological information

Transcriptome data and clinical information for LUAD were gathered from The Cancer Genome Atlas (TCGA) using the Genomic Data Commons download tool.

#### Lung cell type enriched transcriptome

The Human Protein Atlas^36^ shows that the lung-specific proteome has 195 raised genes, 17 enriched genes, and 42 group-enriched genes. The Human Protein Atlas gave us the results acquired from the following links:

https://genemania.org/search/homo-sapiens/pparg.

http://stitch.embl.de/cgi/network.pl?%20taskId=KpczawHjZzrz.

https://thebiogrid.org/111464/summary/homo-sapiens/pparg.html.

#### Systematic data base-mining and enrichment-analyses of PPARG gene expression profile and prognostic role in lung adenocarcinoma (LUAD)

##### UALCAN data base mining

The open-access platform UALCAN (http://ualcan.path.uab.edu/) is based on level 3 RNA-seq and pathological files from the TCGA database^37^ and was used to examine the relative transcriptional levels of the PPARG gene between tumor and non-cancerous tissues, as well as the correlation of PPARG gene mRNA levels with pathological features.

##### Differential expression of PPARG gene in LUAD and normal tissues

We are looking at how much PPARG is expressed in normal tissues compared to tumor tissues using data from the GTEx Portal^38,39^ Release V8, which can be found at this link: https://exphewas.ca/v1/gene/ENSG00000132170?analysis_subset=BOTH#cardio-endpoints-results-section.

https://www.gtexportal.org/home/gene/ENSG00000132170.

http://ualcan.path.uab.edu/cgi- bin/TCGAExResultNew2.pl? genenam = PPARG&ctype = LUAD.

The expression of the PPARG gene in LUAD is shown according to the type of tumor, its stage, whether it has spread to lymph nodes, and the mutation status of the TP53 protein. https://ualcan.path.uab.edu/cgi- bin/TCGAExResultNew2.pl? genenam = PPARG&ctype = LUAD.

The TP53 mutation status was determined using whole exome sequencing data from TCGA, which was obtained via Mutation Annotation Format (MAF) files generated by VarScan2 on the Genomic Data Commons portal. The samples with and without the TP53 mutation were matched to RNA-seq data.

##### GEPIA database mining

GEPIA, an interactive website (http://gepia.cancer-pku.cn/) Using data from the Cancer Genome Atlas and the TCGA database, “expression level analyses”^40^ were conducted to study the prognostic values and survival related to PPARG gene expression in lung cancer and nearby normal tissue samples. The gene expression patterns of the PPARG-correlated genes are also extracted and rated alongside PPARG. We acquired the data results from the following link: https://ualcan.path.uab.edu/cgi-bin/TCGAExHeatMap5KK.pl?%20cantype=LUAD&correlFile=PPARG%23%23QlWdS8a2H.

##### ImmuCellAI analysis

The Immune Cell Abundance Identifier (ImmuCellAI) program, http://bioinfo.life.hust.edu.cn/ImmuCellAI, calculates the abundance of 24 immune cells from gene expression datasets obtained by RNA-Seq and microarray data^41^.

The 24 immune cells are divided into 18 T-cell subtypes and six other types: B cells, natural killer (NK) cells, monocyte cells, macrophage cells, neutrophil cells, and dendritic cells. The 24 immune cells are divided into two layers: innate immunity, which includes DC, B cells, monocytes, macrophages, NK, neutrophils, CD4 T, CD8 T, NKT, and Tgd. Layer 2 of adaptive immunity includes CD4 naive, CD8 naive, Tc, Tex, Tr1, nTreg, iTreg, Th1, Th2, Th17, Tfh, Tcm, Tem, and MAIT.

We obtained the expression data for the 24 immune infiltrating cells corresponding to LUAD samples from the ImmuneCellAI website. The revised database includes an R package for retrieving heatmap and pairwise correlation matrix visualizations. The database application is called Tumor Immune Estimation Resource (TIMER 2.0).

https://cistrome.shinyapps.io/timer/ is a web server that brings together resources for studying immune cell abundance and gene expression in LUAD^42^. We validated the ImmuneCellAI data using the TIMER 2.0 database and found it to be supportive.

The GEPIA database, http://gepia.cancer-pku.cn/, provides correlation curves between PPARG expression levels and immune infiltration of tumor-infiltrating lymphocytes, CD8 + T cells, CD4 + T cells, macrophages, neutrophils, and dendritic cells in LUAC.

#### PPARG protein-protein interaction and functional network

The Protein-Protein Interaction (PPI) network was constructed using GeneMANIA version 3.6.0 http://www.genemania.org/^43^ Utilizing a massive collection of functional association data to predict PPARG gene function and discover additional genes connected to PPARG, BioGRID^4.4^ database https://thebiogrid.org/ and STITCH version: 5.0 http://stitch.embl.de/ Protein and genetic relationships, pathways, co-expression, co-localization, and protein-domain similarity are all association data used to draw the rank showing important interacting molecules. The data obtained from GeneMANIA and STITCH for the PPARG network of predicted functional partners met the results obtained from BioGRID^4.4^ https://thebiogrid.org/111464/summary/homo-sapiens/pparg.html (Accessed April 20th, 2024).

The data could be visualized through these links:

GeneMANIA network: https://genemania.org/search/homo-sapiens/pparg.

STITCH network: http://stitch.embl.de/cgi/network.pl?%20taskId=KpczawHjZzrz.

BioGRID^4.4^ network: https://thebiogrid.org/111464/summary/homo-sapiens/pparg.html (Accessed April 20th, 2024).

### Molecular docking

Polar hydrogens were added to the resulting PDB files^44^, and docking was carried out using the Molecular Operating Environment (MOE) version 2014.0901 software (Chemical Computing Group Inc., Quebec, Montreal, Canada).

https://www.chemcomp.com/Research-Citing_MOE.htm.

We treated the protein to remove repeated chains and water molecules. The MOE QuickPrep methodology was used to refine the structure, 3D protonation, and partial charge calculation using an RMSD gradient of 0.1 kcal/mol and the AMBER10:EHT field.

In this study, we employed rigid docking to calculate the binding affinity scores (KJ/mol) of pioglitazone and telmisartan to albumin and extracellular exosomes, which serve as carrier proteins for targeted lung medicine administration.

For the MyoVc carrier protein, we used the SiteFinder | 3D server’s site finder tool^45^ to find the largest pocket with 57 amino acids, verifying that the tested drugs fit well.

We used the MOE Dock protocol and its descriptors to establish the optimum postures and binding score values for the drugs under investigation. We ran molecular docking with the MOE default settings, using the triangle matcher as the placement method and London dG as the primary scoring function. We added another refinement step by integrating the stiff docking approach with the GBVI/WSA dG affinity score algorithm^46^. Furthermore, the test compound’s first docking pose was the best, with the highest binding energy value. The best pose was finally chosen, photographed, and exported as a JPEG.

### MD simulation of telmisartan in cargo binding domain from human MyoVc

A molecular dynamics simulation of telmisartan was performed for 18 ns, demonstrating the drug’s stability within the cargo-binding region of the human carrier protein MyoVc (PDB code: 4L8T), which is crucial for the exosome’s cargo. Molecular dynamic simulations employed Molecular Mechanics with Generalized Born and Surface Area solvation method (MM/GBSA) to calculate the free energy of ligand binding to proteins (Godschalk et al., 2013).

The initial PDB files for the optimal pose of the telmisartan compound in complex with the protein were prepared for the run. MD simulations were conducted for 19 nanoseconds at 310 K, equivalent to body temperature, utilizing Nanoscale Molecular Dynamics (NAMD 2.14 Release - Aug 2020) software https://www.ks.uiuc.edu/Research/namd/, which is founded on the Charm + + programming model. The pH was kept at a standard physiological level of 7. We employed the all-hydrogen AMBER99SB force field to accurately represent molecular interactions. The geometry of telmisartan was optimized through quantum mechanics (QM) calculations using Gaussian 16 software https://gaussian.com/gaussian16/, and the Schrödinger equation was solved for the molecule^47^. The input consists of the coordinates, net charge, and total spin. The outcome comprises the total energy and the wave function, which serve as the basis for calculating all measurable properties of the system.

The final charges for Telmisartan were assigned to the compound utilizing Antechamber, and the force field parameters adhered to GAFF2 standards^48^. The general AMBER force field is utilized for rational drug design, as detailed on the official website, ensuring precise modeling of molecular forces. The complex was located centrally within a traditional three-point water model, the TIP3P water cube, with dimensions that provided a minimum 14 Å water buffer surrounding the complex. To replicate physiological conditions, the complex was neutralized and adjusted to a 150 mM ionic concentration of sodium and chloride ions, accomplished by replacing water molecules with the highest electrostatic potential at their oxygen atoms with these ions. The current MD protocol commenced with an initial depreciation of the fully solvated and ionized systems, succeeded by a gradual temperature increase to the target level. We imposed substantial constraints on the protein backbones and bound telmisartan to avoid unrealistic modifications during the initial simulation phases. Following heating, the systems experienced a 1 ns equilibration under periodic boundary conditions, during which energy restraints were gradually lifted. The simulations continued for an additional 17 ns, recording atomic coordinates at intervals of 0.1 picosecond for comprehensive analysis. This experimental method for molecular dynamics provided a controlled simulation environment, essential for accurately investigating the dynamics and stability of the human myosin cargo binding domain and telmisartan complex.

## Results and discussion

### Bioinformatics studies

This study is part of a bigger project in our group that uses natural products that have been changed into new uses, like prodigiosin, camptothecin, and hinokitiol, to target different types of cancer^49–51^. The project also uses man-made small-molecule drugs that target signaling pathways in diseases like cancer^52,53^.

Two drugs called pioglitazone and telmisartan^54^ are known to trigger PPARG. This study investigated whether they could help cure lung cancer by using exosomes. Exosomes that cells release could be used to deliver drugs for the focused treatment of certain diseases^55,56^.

Before doing any experiments in cells or living things, we used computer analysis, bioinformatics, cheminformatics, molecular modeling, and simulation to guess how the two PPARG agonist drugs would react and interact with their target molecules in lung cancer, as well as with proteins and drug cargo. This assumption was very important before making the drug-exosomal cargo for the lungs. It made sure that it was only taken up by lung cancer cells so that the drug could be given in a controlled and long-term way to create an “effective, personalized, and targeted” (EPT) treatment.

#### Lung cell type enriched transcriptome

The Human Protein Atlas indicates that the lung has the most group-enriched gene expression similarity to lymphoid tissue, with the PPARG gene being one of the enriched genes expressed in the lung. Table 1 presents the number of genes within each specificity group for the lung cell types.

**Table 1:** Number of genes in each specificity category in the lung cell types.

Lung cell type	Number of enriched genes			Total number enriched
	Very high	High	Moderate	
Respiratory ciliated cells	591	53	37	681
Alveolar cells type 1	43	41	48	132
Alveolar cells type 2	20	122	221	363
Mitotic cells	111	26	16	153
NK-cells	3	13	39	55
B-cells	4	1	6	11
Endothelial cells	0	9	88	97
Smooth muscle cells	14	13	36	63
Fibroblasts	28	64	164	256
Macrophages	4	51	105	160
Neutrophils	61	52	47	160
Mast cells	10	4	3	17
T-cells	6	29	54	89
Plasma cells	166	15	4	185
All cell types	1061	493	868	2422

#### Systematic database mining and enrichment-analyses of PPARG gene expression profile and the prognostic role in lung adenocarcinoma (LUAD)

**Fig. 2:**
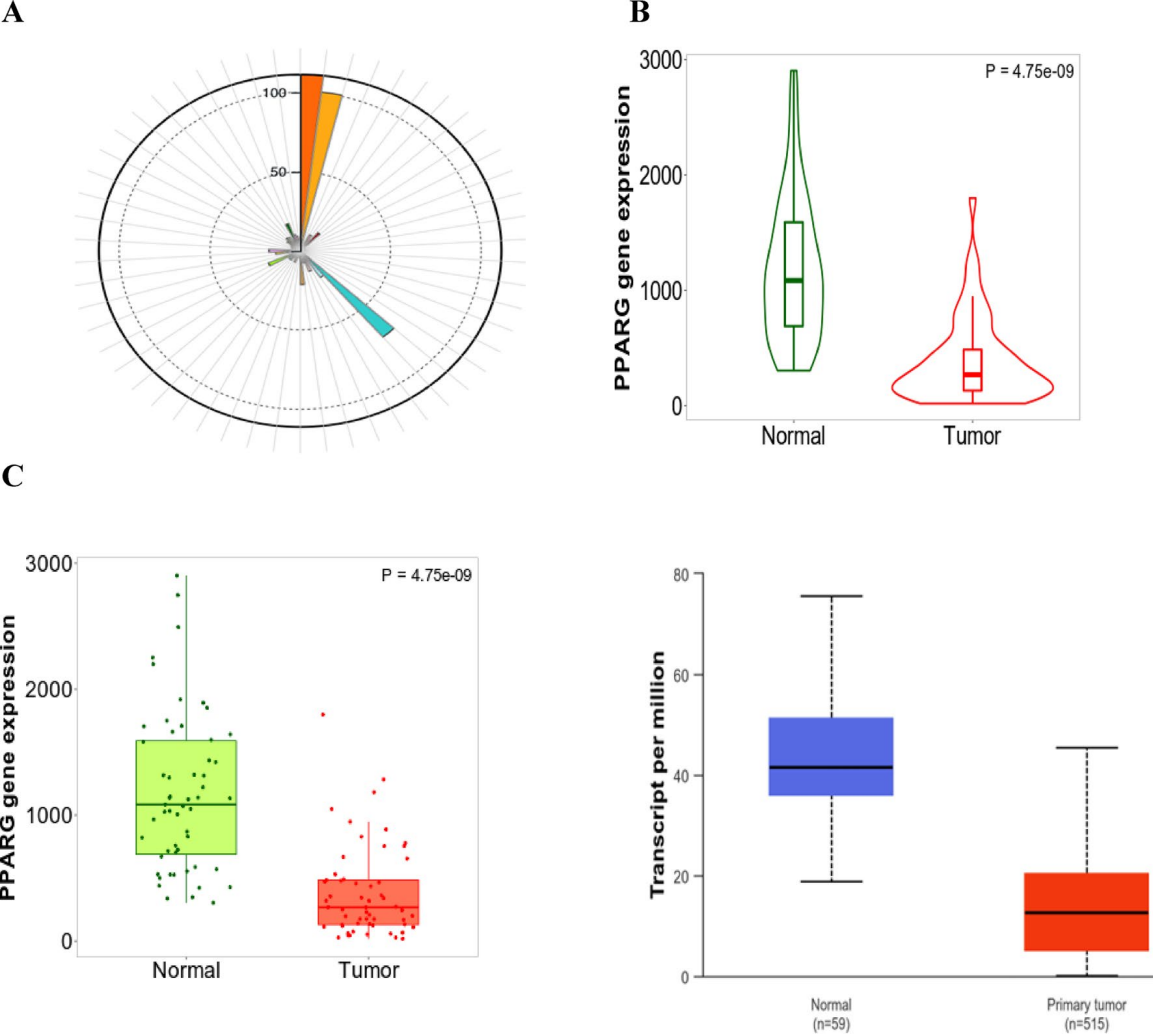
PPARG gene expression based on sample type, from TCGA samples, comparing primary lung tumor (*n* = 515) to 59 normal samples, by UALCAN, showing statistical significance (**A**) median gene-level TPM is 19.5 for the lung (green line pointing to 8 o’clock) for 578 samples. (**B**) violin plot and (**C**) box plot showing the higher expression of the PPARG gene in lung-normal tissue for 59 cases compared to tumor tissue for 515 cases.

Utilizing the UALCAN database^37^ associated with the GTEx database gateway, we examined the expression levels of the PPARG gene in normal and lung cancer cells, revealing that it is expressed at elevated levels in normal tissues relative to malignant tissues **(**Fig. 2**)**.

Figure 2 illustrates PPARG gene expression based on sample type, using TCGA samples to compare primary lung tumors (*n* = 515) with 59 normal samples, as analyzed by UALCAN, and indicates statistical significance (A). The median gene-level TPM is 19.5 for the lung (green line pointing to 8 o’clock) for 578 samples. (B) The violin plot and (C) the box plot illustrates that the PPARG gene is expressed at higher levels in lung-normal tissue from 59 cases compared to tumor tissue from 515 cases.

**Fig. 3:**
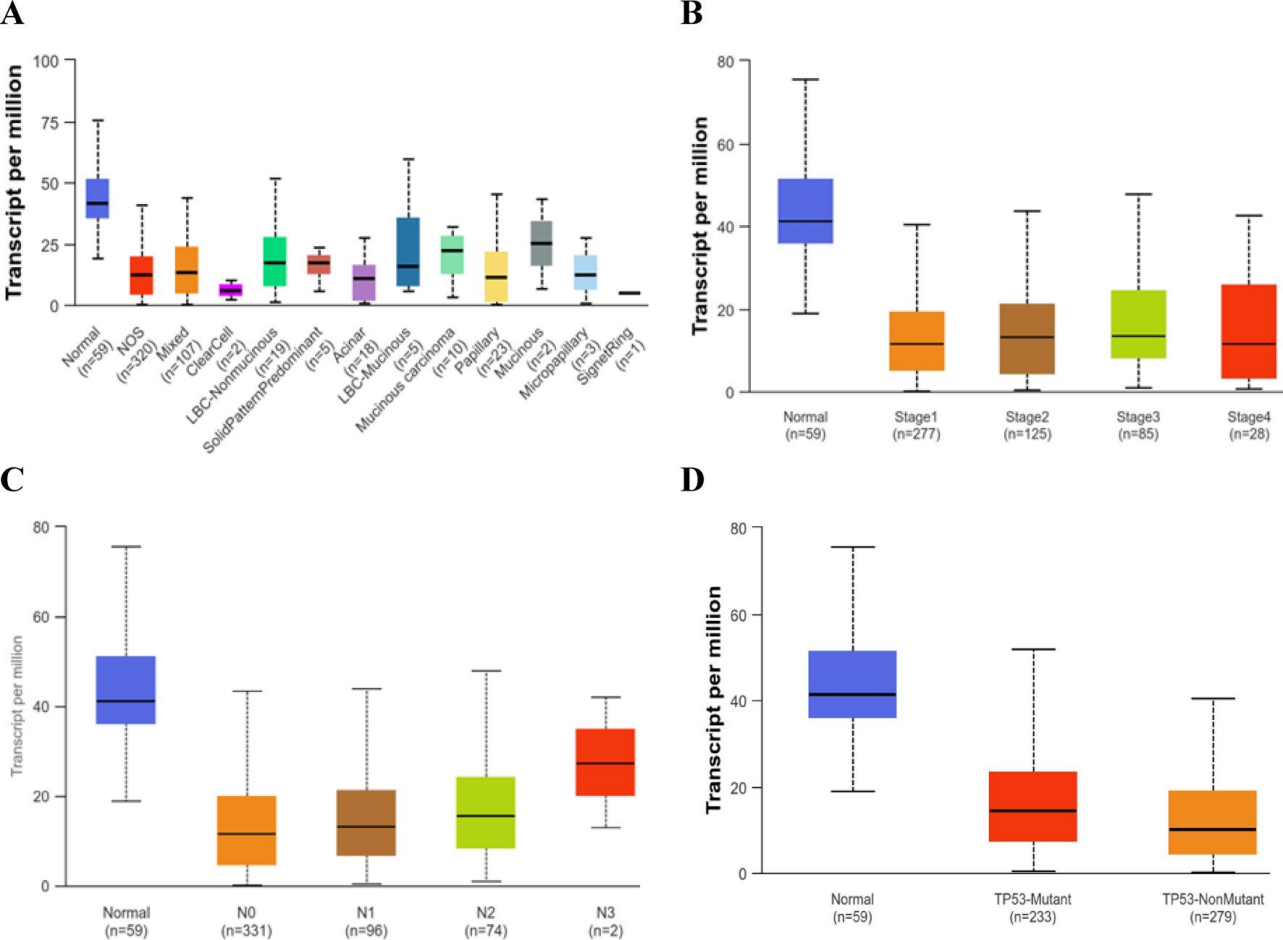
PPARG gene Expression in transcript/million in LUAD based on histological subtypes (**A**), individual cancer stages (**B**), nodal metastasis (**C**), and TP53 mutation status (**D**), from TCGA samples. [NOS: Lung Adenocarcinoma-Not Otherwise Specified, Mixed: Lung Adenocarcinoma Mixed subtype, Clear Cell: Lung Clear Cell Adenocarcinoma, LBC-Non mucinous: Lung Bronchioloalveolar Carcinoma Non-mucinous, Solid Pattern Predominant: Lung Solid Pattern Predominant Adenocarcinoma, Acinar: Lung Acinar Adenocarcinoma, LBC-Mucinous: Lung Bronchioloalveolar Carcinoma Mucinous, Mucinous: Mucinous (Colloid) Carcinoma, Papillary: Lung Papillary Adenocarcinoma, Mucinous: Lung Mucinous Adenocarcinoma, Micropapillary: Lung Micropapillary Adenocarcinoma, Signet Ring: Lung Signet Ring Adenocarcinoma. N0: No regional lymph node metastasis, N1: Metastases in 1 to 3 axillary lymph nodes, N2: Metastases in 4 to 9 axillary lymph nodes, N3: Metastases in 10 or more axillary lymph nodes, nodal metastasis is not available for 1 sample, NX: cancer in nearby LN cannot be measured in 11 samples.].

PPARG gene expression plots **(**Fig. 3**)** based on tumor histology **(**Fig. 3A**)**, tumor stage **(**Fig. 3B**)**, nodal metastasis **(**Fig. 3C**)**, and tissue TP53 mutation status **(**Fig. 3D**)** verified the significantly higher levels of gene expression in normal lung cells and tissues compared to the different histological features of LUAC and the lung tumor tissues at stages 1–4, respectively. PPARG gene expression was consistently higher in normal cases than in LUAC cases, regardless of whether they had absent or positive lymph nodal metastases and whether their TP53 status was non-mutated or mutated. The data presented in this phase of bioinformatics also demonstrates the role of PPARG gene activation in lung tissue protection against LUAC proliferation and metastases.

Figure 3. is using TCGA samples, we assessed the transcript/million of the PPARG gene in LUAD according to histological subtypes (A), individual cancer stages (B), nodal metastasis (C), and TP53 mutation status (D). [NOS: Lung Adenocarcinoma—Not Otherwise Specified; Mixed: Lung adenocarcinoma Mixed subtype with transparent cells: lung The condition is known as clear cell adenocarcinoma. LBC Nonmucinous: Lung Bronchioloalveolar Carcinoma. A solid pattern in the lung is a defining feature of non-mucinous adenocarcinoma. Acinar: Lung. Acinar adenocarcinoma, LBC-mucinous: lung bronchoalveolar carcinoma. Mucinous (colloid) Carcinoma Papillary: Lung papillary adenocarcinoma. Mucinous: Lung Mucinous adenocarcinoma. Micropapillary: Lung micropapillary adenocarcinoma. Signet Ring: Lung. Signet Ring Adenocarcinoma. N0: No regional lymph node metastasis; N1: Metastases in 1 to 3 axillary lymph nodes; N2: Metastases in 4 to 9 axillary lymph nodes; N3: Metastases in 10 or more axillary lymph nodes; nodal metastasis is not available for one sample. NX: Eleven samples failed to detect cancer in the neighbouring LN.

**Fig. 4:**
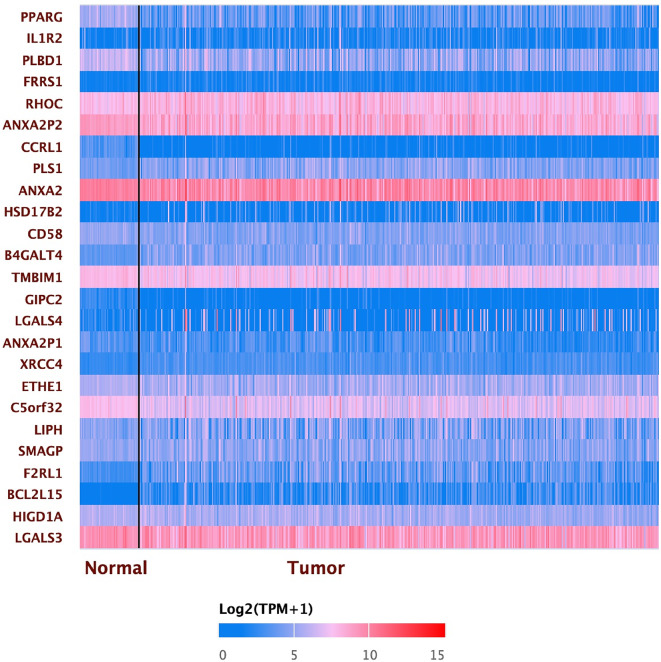
Heatmap of the expression pattern of PPARG and other input genes in LUAD is positively correlated to the PPARG gene.

The heatmap of PPARG gene expression in LUAC and other related genes revealed that PPARG is the most important gene associated with LUAC’s poor prognosis, particularly when compared to normal tissue **(**Fig. 4**)** from the GEPIA database.

Additionally, ImmuneCellAI^41^ is used to gather expression data from 24 types of immune infiltrating (TIF) cells in LUAD samples to find and categorize the TIF cell subtype that has strong links to LUAC.

**Fig. 5:**
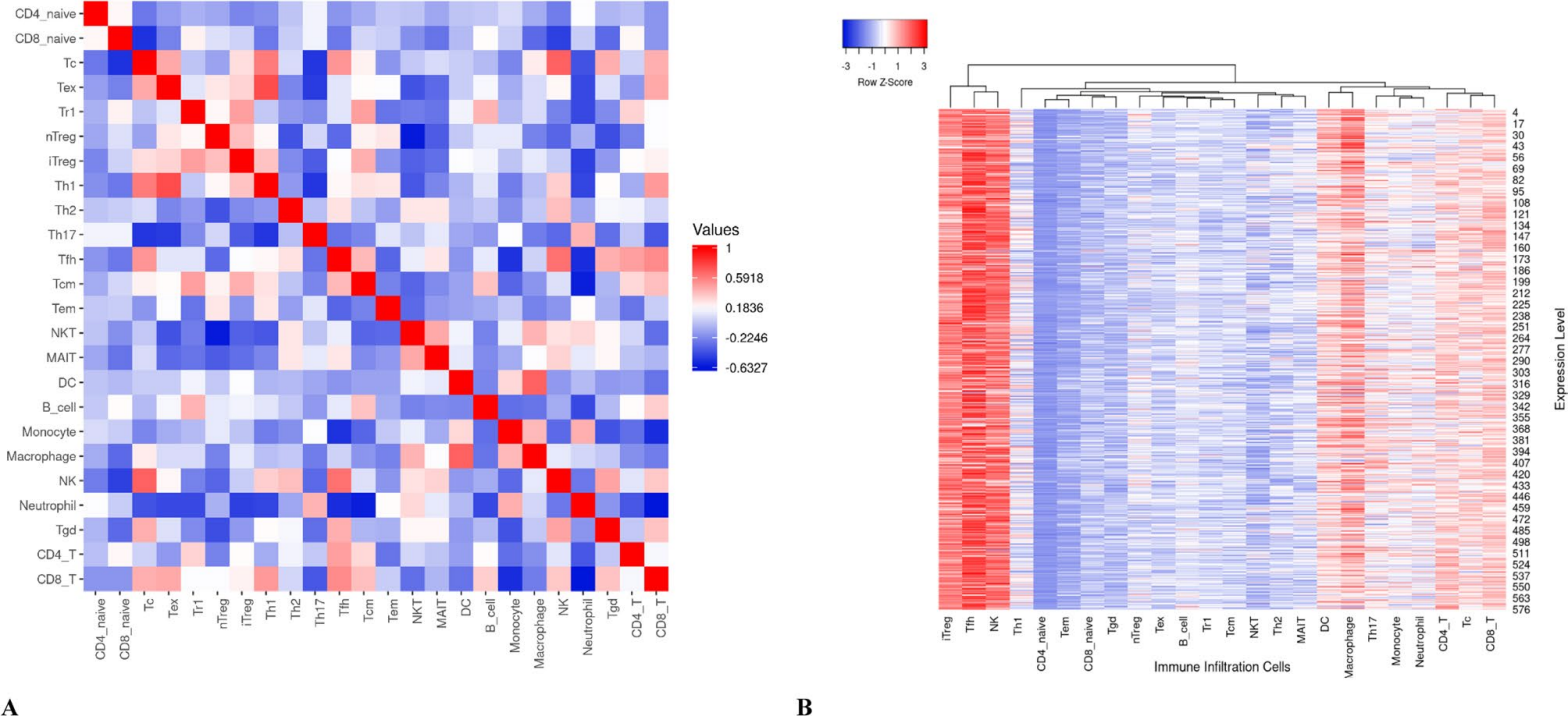
Different immune infiltrating cell expression levels in LUAD samples retrieved from ImmuneCellAI. (**A**) Pairwise correlation values matrix for exploring the immune infiltration cells abundance in 576 samples, and (**B**) Heatmap presentation of the immune infiltrating cell expression levels in LUAC.

 The new database includes an R tool for retrieving the pairwise correlation matrix (Fig. 5A) and heatmap **(**Fig. 5B**)** visualizations. Furthermore, examining the immune infiltration cell abundance in 576 ImmuneCellAI samples **(**Fig. 5B**)** revealed high levels of expression of macrophages, NK, DC, and other T-cell subtypes, which is corroborated by results from the TIMER 2.0 database program^42^. Additionally, the pairwise correlation matrix showed a strong connection between T-cell subtypes, macrophages, and DCs **(**Fig. 5A**)**.

**Fig. 6:**
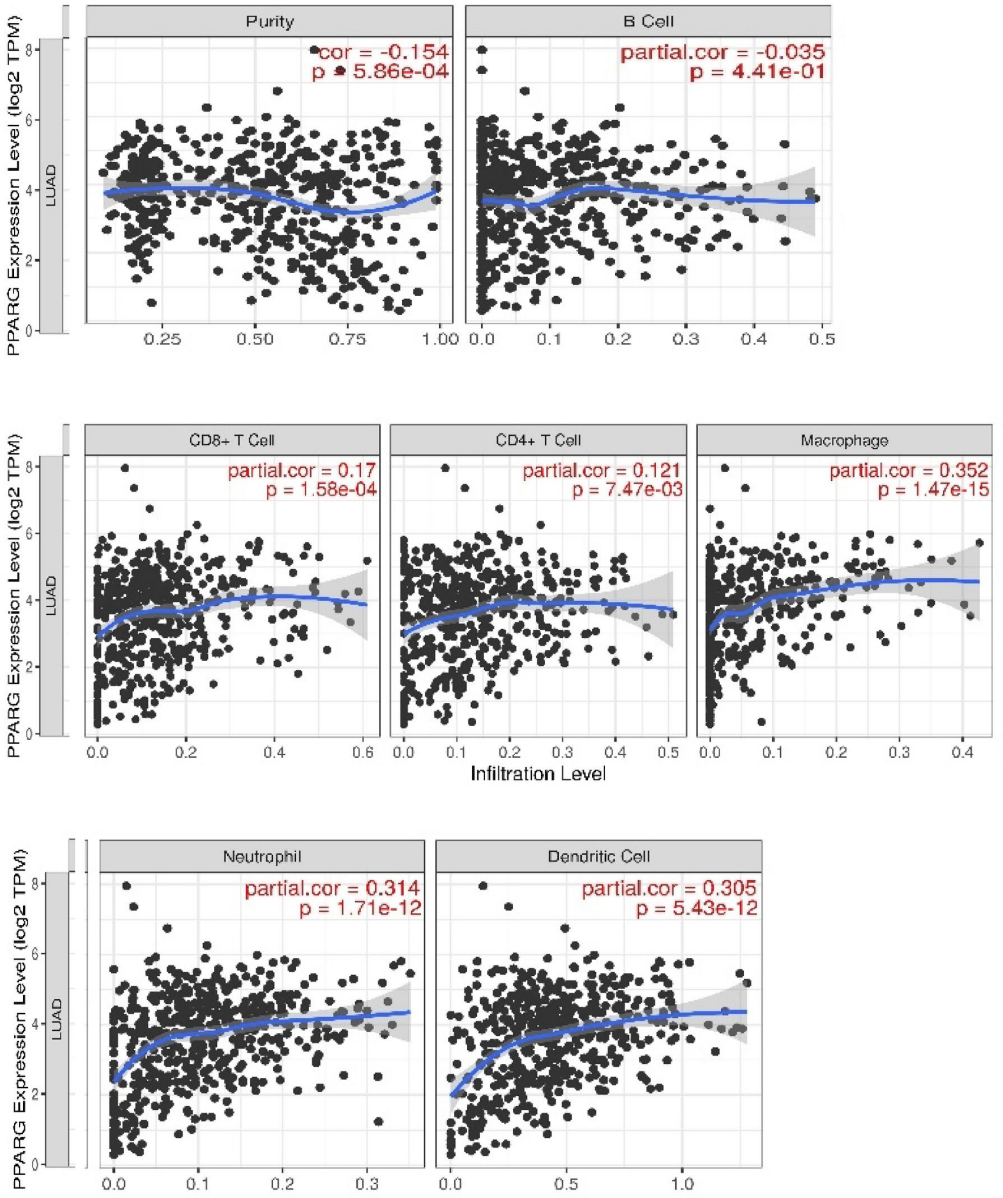
Correlation values of PPARG gene expression level (expressed as log2 TPM) with the tumor immune infiltration cells level in LUAD.

Accordingly, we studied the correlation of the PPARG gene expression level to the tumor-infiltrating lymphocytes, CD8+, CD4+, macrophages, neutrophils, and dendritic cells in LUAC as an excellent sign for cancer disease prognosis^57^. Surprisingly, the gene expression was negatively correlated to B cells and positively correlated to CD8 + and CD4 + subtypes of T-lymphocytes in terms of the partial correlation values (Spearman’s rho) obtained from the GEPIA database **(**Fig. 6**)**.

To better understand how the PPARG gene functions in LUAD, we examined critical genes that interact with it via gene-protein linkages and gene-gene links, as well as creating and analyzing a PPI network.

**Fig. 7:**
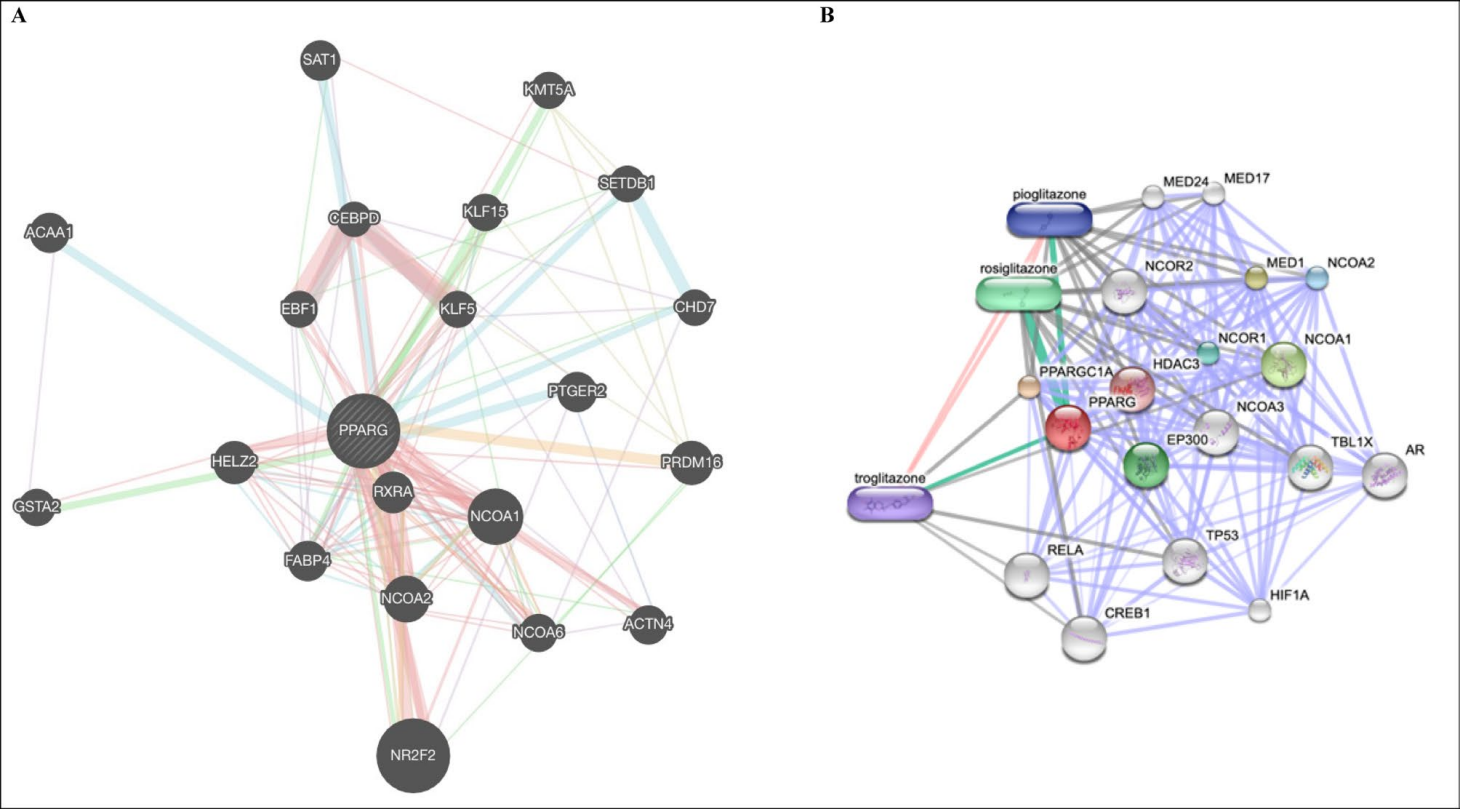
(**A**) The GeneMANIA presentation of PPARG’s protein-protein interaction (PPI) network shows networks for physical interactions between genes as pink lines, co-expression as violet lines, predictions as yellow lines, common route pathways as light blue lines, and genetic interactions as green lines. Additionally, shared protein domains were considered when examining these protein relationships. (**B**) The STITCH presentation of PPARG predicted functional partners’ networks.

Therefore, drugs such as pioglitazone and telmisartan can stimulate the PPARG protein, potentially aiding in the destruction of cancer cells^58^ either by affecting genes or through pathways related to PPARG that were identified via computer analysis (Fig. 7).

### Cheminformatics studies of the repurposed Pioglitazone and Telmisartan

#### In-silico measurement of physicochemical properties and Tox21^59^

We used the Admetlab 2.0 online tool to assess the physicochemical features of pioglitazone and telmisartan, such as logP (lipophilicity) and TPSA (topological polar surface area). We also investigated how well the drugs bind to plasma proteins, their ability to cause breathing problems, their effects on mitochondrial membrane potential, aromatase enzyme activity, aryl hydrocarbon receptor activity, human liver toxicity, and liver injury caused by drugs used to treat the same drugs.

According to the results in Table 2, pioglitazone (logP = 3.01) and telmisartan (logP = 6.249) are both effective in mixing with fats, but telmisartan performs better. The TPSA values for pioglitazone (68.290 Å²) and telmisartan (72.940 Å²) indicate that these drugs can mix with lipids, but not adequately enough to pass through the blood-brain barrier, keeping them safe from possible side effects in the central nervous system. The TPSA values of pioglitazone (68.290 Å²) and telmisartan (72.940 Å²) revealed that these test drugs are lipophilic but not adequate to penetrate the blood-brain barrier (BBB), making them safe regarding any potential central nervous system (CNS) side effects^62,63^. These findings also demonstrated that telmisartan and pioglitazone may be employed efficiently in targeted delivery systems based on various fat-loving materials, such as albumin and lung exosomes (Lung-Exo), without producing any physical problems.

Based on computer estimates of how dangerous compounds are from the Tox21 Data Challenge, which is part of the NIH and FDA’s “Toxicology in the 21 st Century” initiative, we determined that pioglitazone, like telmisartan, affects SR-MMP activity (Table 2). Notably, investigations have demonstrated that pioglitazone causes mitochondrial damage, supporting the predictions **(**Table 2**)**. Interestingly, experimental evidence^64^ reveals that pioglitazone’s mitochondrial toxicity fits expectations **(**Table 2**)**. The test compounds demonstrated activity on NR-Aromatase and NR-AhR, both of which are part of the TOX21 pathway. The projected results **(**Table 2**)** indicated that pioglitazone and telmisartan had the potential to cause hepatotoxicity and drug-induced liver damage. Some experimental research^65,66^ demonstrated pioglitazone’s ability to generate hepatic energy alterations, which validated the data given in Table 2.

**Table 2:** The predicted physicochemical property and TOX21 of Pioglitazone and Telmisartan.

Parameter	Pioglitazone	Telmisartan	Comment
**LogP (W/O)**	3.021	6.249	Optimum
**TPSA (Å** ^**2**^ **)**	68.290	72.940	Lipophilic
**PPB**	97.171%	98.951%	Strong binder
**Respiratory Toxicity**	--	--	Non-toxic
**SR-MMP**	++	++	Active
**NR-Aromatase**	+++	++	Active
**NR-AhR**	+	+	Active
**H-HT**	++	+++	Hepatotoxic
**DILI**	+++	+++	Hepatotoxic

[LogP: partition coefficient, o/w: octanol/water, TPSA: topological polar surface area, PPB: Plasma Protein Binding, SR-MMP: Mitochondrial Membrane Potential, NR-AhR: nuclear receptor-aryl hydrocarbon receptor, H-HT: human hepatotoxicity, DILI: drug-induced liver injury.].

#### Pioglitazone and Telmisartan biological targets of the highest corresponding probabilities

Using the Swiss Target Predictor^67,68^, we discovered that pioglitazone and telmisartan are likely to interact with various biological targets, including receptors, enzymes, and transporter proteins **(**Table 3**)**. Both drugs were shown to influence the liver bile salt export pump, the kidney enzyme carbonic anhydrase II (CA2), and the lung angiotensin-converting enzyme (ACE). The interactions match the known harmful effects of pioglitazone^65,66^ and telmisartan^69^ along with the expected information from Admetlab 2.0^60,61^ about liver toxicity and damage **(**Table 2**)**. The predictor determined that both drugs target the type-1 angiotensin II receptor (AGTR1) **(**Table 3**)**. The findings imply that pioglitazone should be investigated for its potential as an AGTR1 binder, which could shed light on the structural properties that allow both telmisartan and pioglitazone to have the pharmacological effects of PPARG agonists and possibly AGTR1 antagonists.

**Table 3:** Predicted Pioglitazone and Telmisartan biological targets of the highest probability.

Drug	Target	Common Name	CHEMBL ID
**Pioglitazone**	PPARG	Peroxisome proliferator-activated receptor gamma	CHEMBL235
	MAOB	Monoamine oxidase B	CHEMBL2039
	CA2	Carbonic anhydrase II	CHEMBL205
	AGTR1	Type-1 angiotensin II receptor	CHEMBL227
	PPARA	Peroxisome proliferator-activated receptor alpha	CHEMBL239
	ABCB11	Bile salt export pump	CHEMBL6020
**Telmisartan**	ACE	Angiotensin-converting enzyme	CHEMBL1808
	AGTR1	Type-1 angiotensin II receptor	CHEMBL227
	PPARG	Peroxisome proliferator-activated receptor gamma	CHEMBL235
	AGTR2	Angiotensin II receptor	CHEMBL4607
	GLRA1	Glycine receptor subunit alpha 1	CHEMBL5845

#### Prediction of Pioglitazone-PPARG, Telmisartan-PPARG nuclear receptor affinity and ligand efficiency

The Zinc20 database^70,71^ provided numerical values for how well pioglitazone (pKi = 7.06) and telmisartan (pKi = 5.82) bind to their targets, supporting earlier findings that classify pioglitazone as a PPARG agonist and telmisartan as a partial agonist^23^. Additionally, the ligand efficiency values from the database show the binding strengths of pioglitazone (0.41) and telmisartan (0.21).

**Table 4:** Predicted Pioglitazone and Telmisartan binding affinity and ligand efficiency.

Drug	Gene Name	Class	pKi (L.E.)
**Pioglitazone**	PPARG (agonist)	Transcription factor/NR	7.06 (0.40)
**Telmisartan**			5.82 (0.21)

The cheminformatics data in this study for pioglitazone and telmisartan highlights the need to create these helpful drugs in lung-derived extracellular vesicles (Lung-Exo) or inhalable exosomes for targeted delivery to lung cells, to reduce the known side effects of the repurposed drugs, and to enhance their effectiveness.

### Affinity and stability studies of pioglitazone and telmisartan into the protein carrier vehicle used for lung-targeted delivery

Molecular docking and dynamics (MD) simulations are used to evaluate how well the repurposed drugs stick to their carrier vehicles and to check if the complex remains stable as it travels from where it is given to where it works.

#### Molecular docking studies of pioglitazone and telmisartan into the carrier vehicle used for lung-targeted delivery

Albumin has been selected as a universal carrier for hydrophobic pharmaceuticals. The diverse human cargos act as vesicles that associate with the canonical non-muscle Myosin V (MyoV) carrier, specifically its paralog VC (MyoVC) or Myo5C, which is involved in cell membrane trafficking and the intracellular transport of cargos, including exosomes, according to KEGG.

To evaluate the binding affinity scores of pioglitazone and telmisartan to human serum albumin and extracellular exosome carrier protein for targeted pulmonary drug delivery, the albumin protein carrier was sourced from the RCSB Protein Data Bank (RCSB PDB) under PDB code 1HK1, which represents human serum albumin (HSA) complexed with thyroxine (3,3’,5,5’-tetraiodo-l-thyronine)^72^. The cargo binding domain from human MyoVC was obtained from the Peptide Atlas^73^ with PDB code 4L8T, which does not contain a crystallized ligand^74^.

We identified the optimal location within the cargo-binding domain of the transporter protein to accommodate the tested pharmaceuticals, enabling an assessment of their compatibility following docking simulations. We selected the pocket with the highest amino acid content and largest dimensions to ensure compatibility with the shape of the target medicines for docking. The pocket selected exhibited the highest amino acid count and largest dimensions, thereby ensuring compatibility with the contoured surface of the target medicines for docking. Conversely, we incorporated these two medications directly into the albumin carrier to facilitate the targeted delivery of thyroxine (T4) within the body.

**Fig. 8:**
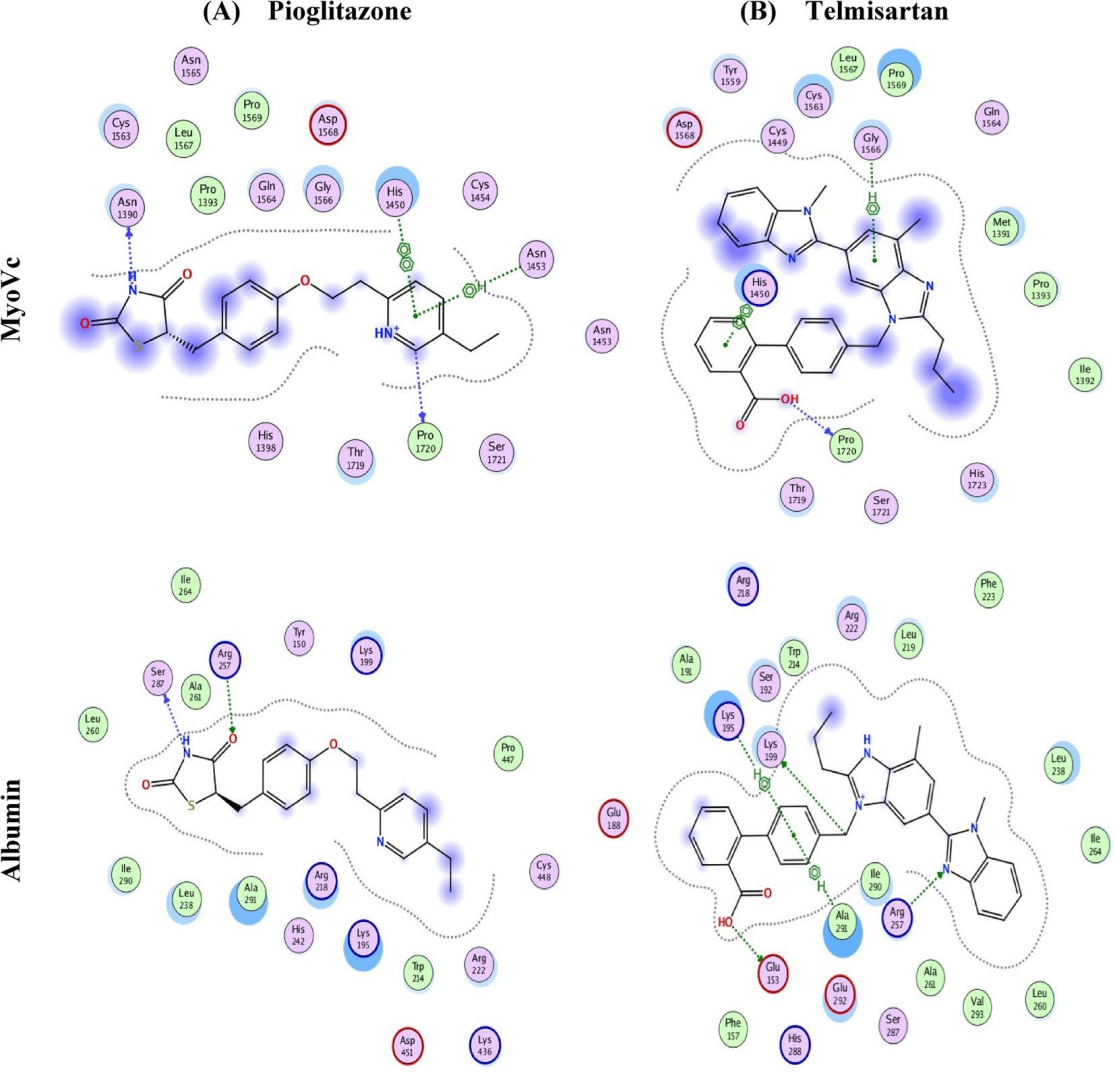

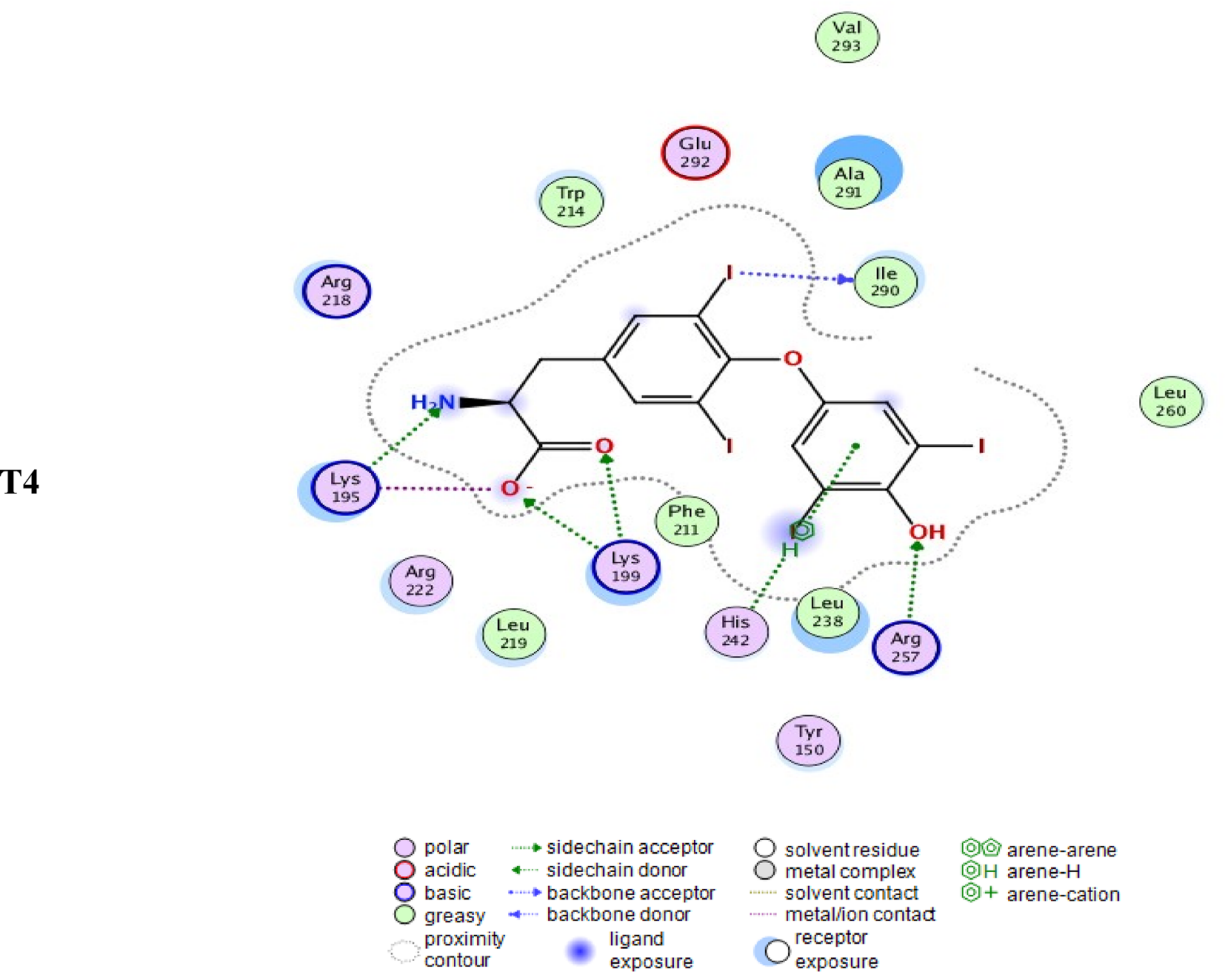
Affinity in complex with Cargo domain of MyoVc (pdb entry 4L8T) vesicles (exosomes) (upper panel) and in complex with albumin (pdb entry 1HK1) (middle panel) compared to albumin carrying T4 (the original ligand) (lower figure). Blue and green dotted arrows and green dots present hydrophobic interactions and hydrogen bonding forces.

The docking results demonstrated that both pioglitazone and telmisartan exhibit strong binding to MyoVc and albumin carriers (Fig. 8), indicating that their interactions with the amino acids at the binding sites of these proteins contribute to the observed high-affinity scores. The findings demonstrate that both drugs effectively treat lung cancer, necessitating reduced doses and less frequent administration. Additionally, the human MyoVc cargo-binding domain transporter can deliver telmisartan to lung tissues for cancer therapy. Our findings indicate that pioglitazone exhibits weaker binding and attraction at the albumin site, suggesting it is less stable compared to telmisartan.

**Fig. 9:**
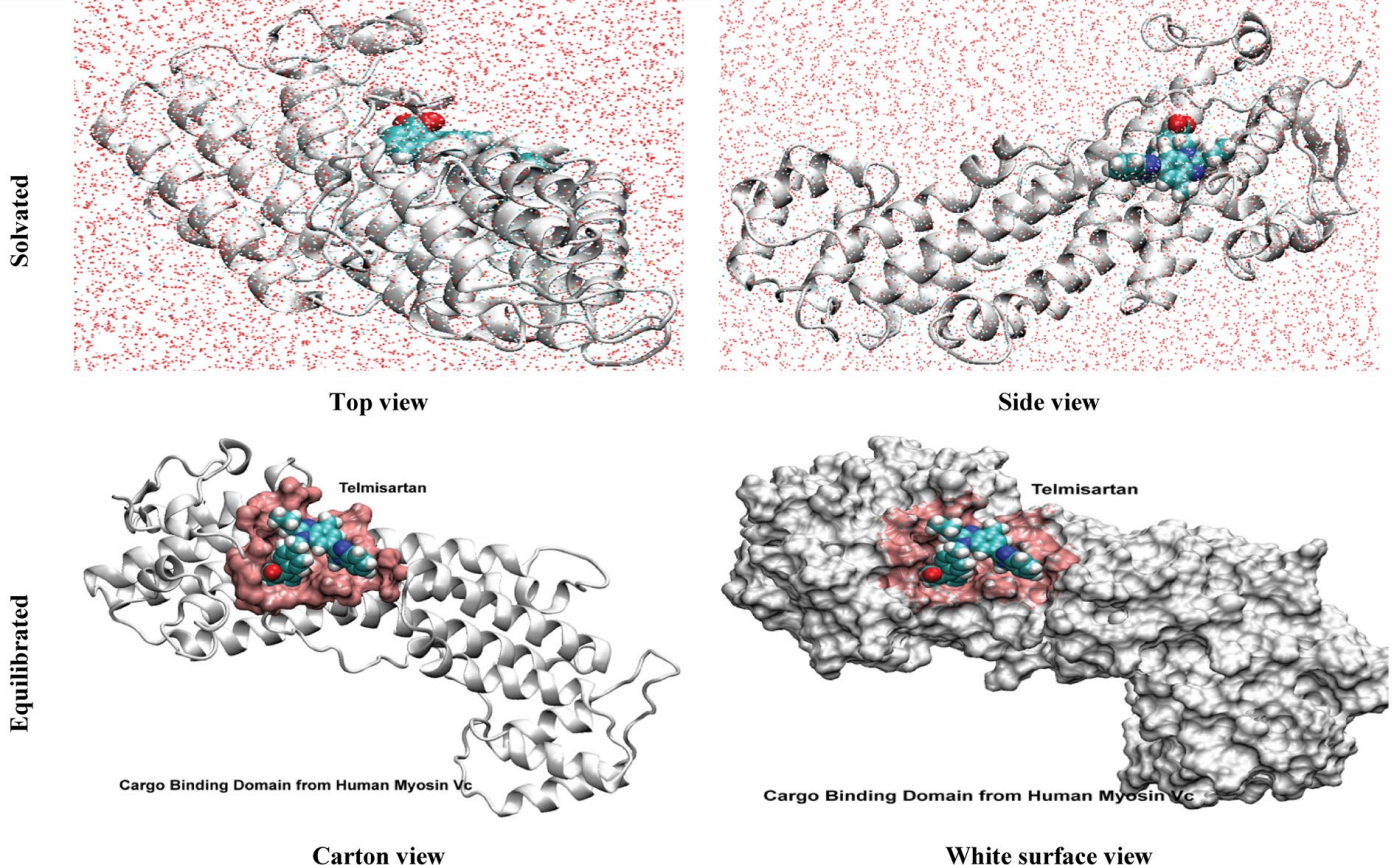
Human MyoVc Cargo Binding Domain and Telmisartan Solvated (upper panel) complex top and side views and equilibrated complex (lower panel) shown in a carton view or with a white surface view with Telmisartan in blue within the pink binding pocket.

Figure 9 presents 2D-style docking solutions for pioglitazone, telmisartan, and T4 within the cargo domain of MyoVc (PDB entry 4L8T) vesicles (exosomes) in the upper panel and albumin (PDB entry 1HK1) in the middle panel, alongside albumin complexed with T4 (the original ligand) in the lower panel. The three-letter and digit protein sequence code denotes amino acid residues, whereas the green and blue dotted lines classify contact forces following the figure’s descriptive scheme.

#### MD simulation of telmisartan in complex with cargo binding domain from human MyoVc

We ran molecular dynamics simulations of the telmisartan-human MyoVc cargo-binding domain complex to study how the protein behaves and changes at the atomic level for 18 nanoseconds. The intricate system reached equilibrium within 1 ns, demonstrating stable atomic fluctuations within 17 ns of the simulation’s duration. This verifies that the simulation time was adequate for the aims of the present investigation, designated as a “TDDS study^75^”.

**Solvation and Equilibration of the Complex**

Figure 9 presents a comprehensive structural analysis of the protein-ligand complex, showcasing the solvated human MyoVc cargo binding domain in conjunction with the Telmisartan complex, along with the equilibrated states of the protein-ligand complex collected at different phases of the simulation. The subsequent perspective of the complex illustrates various approaches to examining the simulated conditions of the protein-ligand complex (MyoVc-telmisartan).

**Root-Mean Square Deviations (RMSD)**

**Fig. 10:**
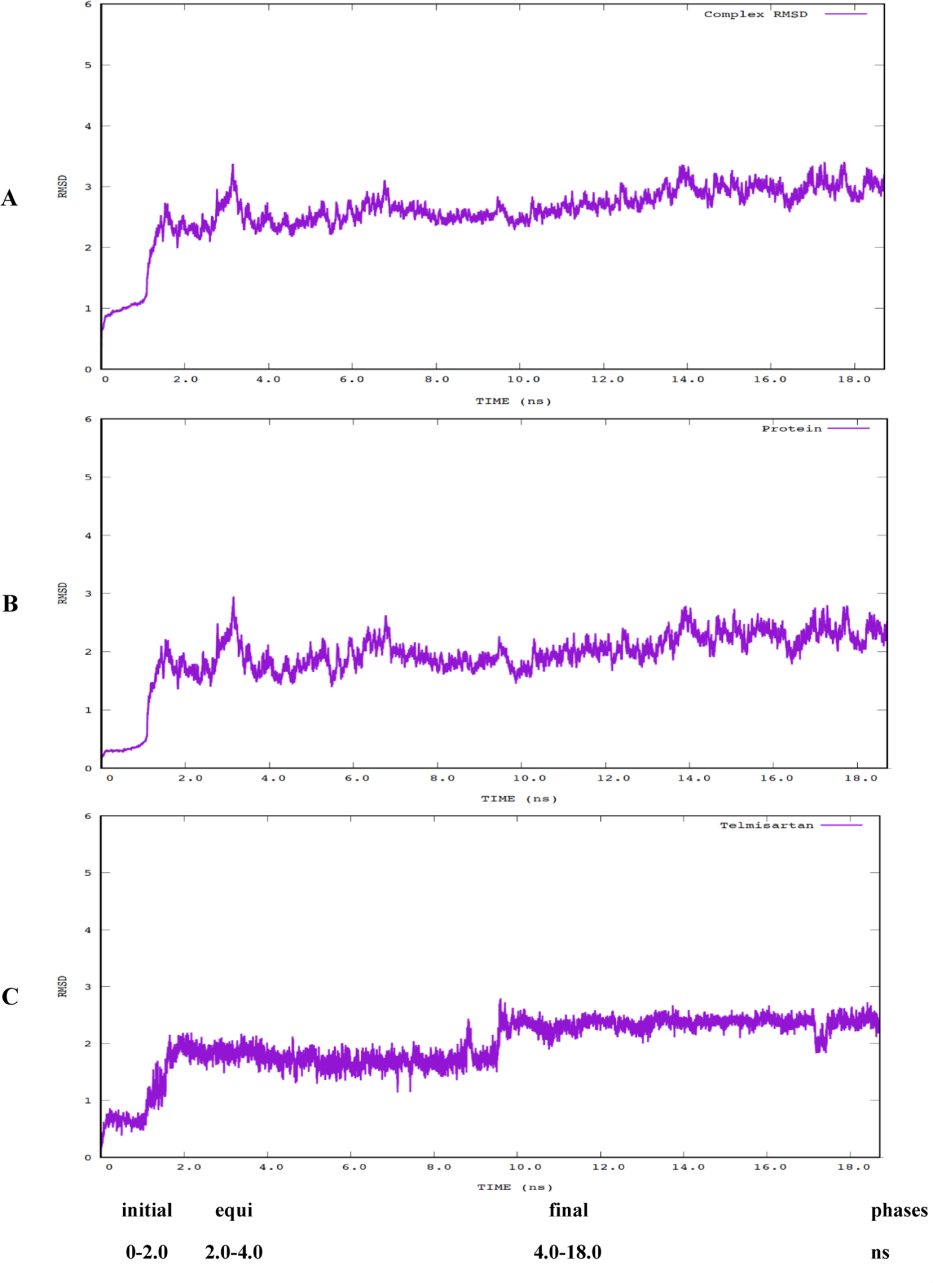
The RMSD graph for the human (**A**) MyoVc Cargo Binding Domain and Telmisartan complex, (**B**) MyoVc Cargo Binding Domain protein only, and (**C**) Telmisartan.

An essential component of our research entailed examining the stability and conformational alterations of the complex, protein, and ligand separately. Figure 10 displays the RMSD values for the complex, protein, and ligand, respectively. These graphs illustrate three separate stages in the execution of molecular dynamics simulations.

The initial phase, lasting 2 ns, includes 50,000 steps of energy minimization. A 0.5 ns duration of incremental heating elevates the system to ambient temperature. We then utilized a 1 ns equilibration phase to steady the system. The concluding step of our simulation method was a prolonged (17 ns) duration of production molecular dynamics simulations, during which the most notable dynamic characteristics of the complex were observed and assessed.

The RMSD analysis of MD simulations for the protein transporter complex **(**Fig. 10A**)**, apoprotein **(**Fig. 10B**)**, and ligand **(**Fig. 10C**)** showed that telmisartan can likely stay attached to the human MyoVc cargo-binding domain at the specific pocket shown in Fig. 8.

**Beta Factor Analysis of Protein Backbone Atoms**

**Fig. 11:**
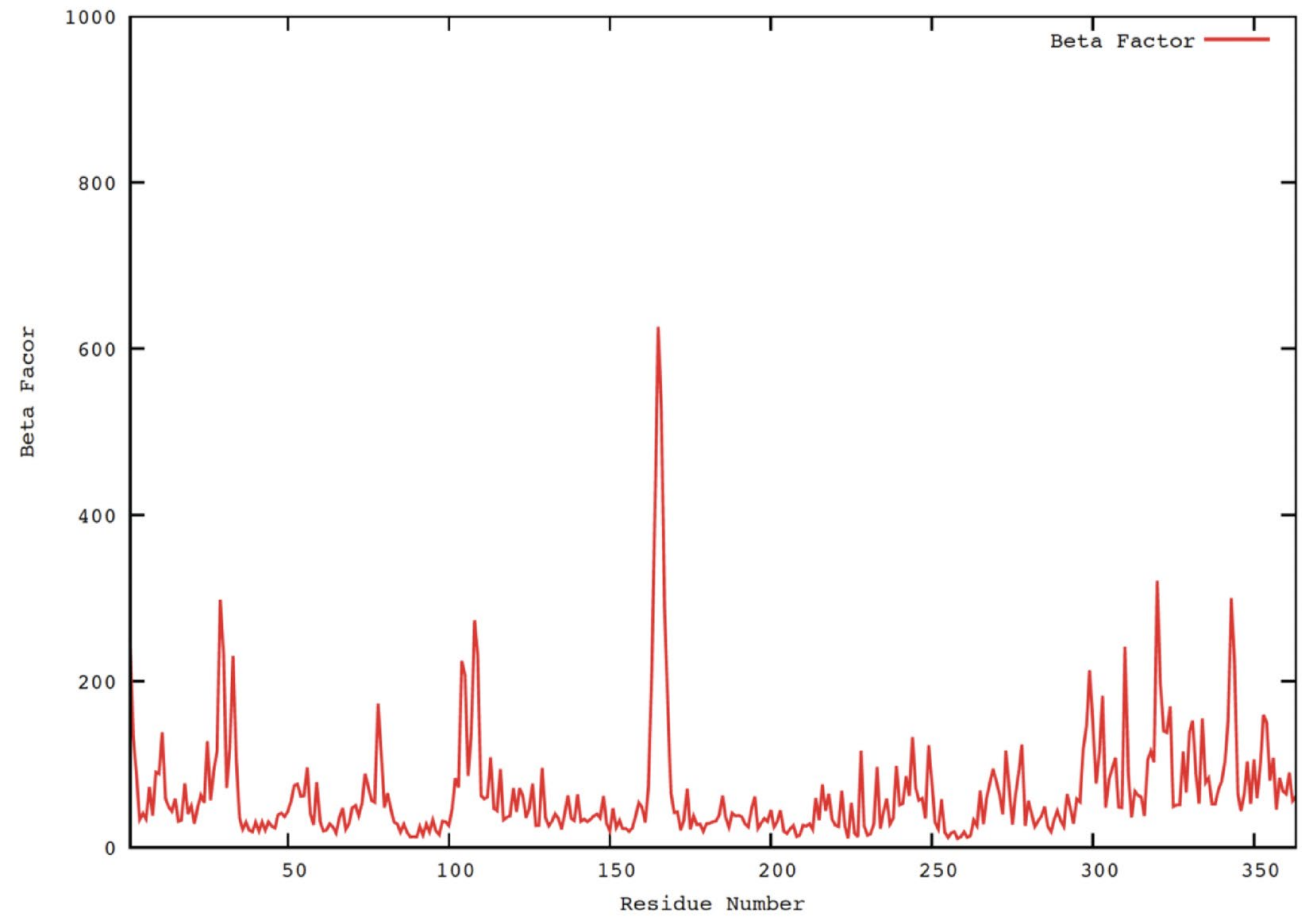
The average atomic fluctuations (Beta Factor) of the Human MyoVc Cargo Binding Domain protein during the molecular dynamic simulations.

To demonstrate the flexibility of the protein backbone atoms and the interactions of telmisartan with the human MyoVc cargo-binding domain within the binding site^76^, we assessed the beta factor of the protein complex throughout molecular dynamics simulations concerning the positions of the protein residues^77^. The highest beta factor values, illustrated in the beta factor plot **(**Fig. 11**)**, were observed at residue numbers 155–180 (auth: 1450–1475), indicating concordance with the contact force established with His 155 (auth: His 1450), as represented by the extracted docking solution of Telmisartan **(**Fig. 8**)**. Additionally, various protein residue numbers exhibited significant changes in atomic motion during the simulation compared to the initial atomic trajectories at 30–40 (auth: 1325–1335), 105–115 (auth: 1400–1410), and 300–345 (auth: 1595–1640). The graphical representation of the beta factor for the protein complex **(**Fig. 11**)** confirmed the flexibility of the protein transporter’s active site in the presence of telmisartan and its atomic interactions. Previous research has investigated the anti-lung cancer effects of TZDs in clinical settings; however, the most effective PPARG agonist, rosiglitazone, exhibits specific undesirable effects, unlike pioglitazone. The side effects of the partial PPARG agonist, telmisartan, are infrequent; however, its gene expression profiles confer superior anti-tumor benefits. In the present study, telmisartan was recognized as an appropriate candidate for the “Beta Factor Analysis” of the human MyoVc cargo binding domain protein during molecular dynamics simulations, which elucidated the stability and flexibility of the MyoVc protein-drug complex and its interaction dynamics with telmisartan.

## Conclusion

The activation of the PPARG transcription factor is linked to reduced NSCLC growth. Research indicates that pioglitazone and telmisartan are potential therapies for NSCLC due to their PPARG-agonistic properties. There is a critical need for new targeted drug delivery formulations for these drugs to minimize systemic toxicity. The study suggests using extracellular vesicles from lung tissue for targeted drug delivery to pulmonary cancer cells. Bioinformatics and cheminformatics data support pioglitazone and telmisartan as promising repurposed drugs for LUAC, highlighting their lipophilicity and compatibility with exosomal components like albumin. Cheminformatics also pointed out potential off-target effects and hepatotoxicity, emphasizing the importance of exosomal targeted delivery. Molecular docking and MD simulations confirmed the affinity and stability of drug-exosomal vehicle complexes. The proposed engineering of exosomal cargo for targeted delivery of these drugs to lung cells could enhance NSCLC treatment and address drug resistance while minimizing systemic toxicity. Finally, the study’s computational technique saves the researchers time and money when building formulations containing biological components for targeted drug delivery.

Nonetheless, experimental validation is on the way as the sustainability of the current manuscript, to emphasize the success of the suggested formulation based on computational work (a limitation).

**Recommendation.** Repurposing PPARG-agonist drugs with or without co-supplementary vitamins with tumor-modulatory effects and positive impact on various disease metabolic/inflammatory pathways as vitamin D^78^ would be crucial point to study as well.

## Data Availability

“No data is generated, all data are incorporated within the manuscript, for any queries the corresponding authors to be contacted”.

## Abbreviations

ABCB11: Bile salt export pump
ACE: Angiotensin-converting enzyme
ADMET: Absorption, distribution, metabolism, excretion, and toxicity
AGTR1: Angiotensin II receptor 1
AGTR1B: Angiotensin II receptor 1B
AGTR2: Angiotensin II receptor 2
AMs: Adhesion molecules
Ang2: AngII: Angiotensin II
ARB: Angiotensin II Receptor Antagonist
AT1: Angiotensin II type 1 receptors
BBB: Blood brain barrier
BP: Blood Pressure
CA2: Carbonic anhydrase 2
CF: Cystic Fibrosis
COPD: Chronic Obstructive Pulmonary Diseases
DB: Database
DC: Dendritic Cells
DDPD: Digital drug property database
DDS: Drug Delivery Systems
ECM: Extracellular matrix
ECVs: Extracellular vesicles
EPT: Effective-Personalized-Targeted
GBSA: Generalized Born and Surface Area solvation
GFs: Growth factors
GLRA1: Glycine receptor subunit alpha-1
H-bonds: Hydrogen bonds
HAS: Human serum albumin
HSP90: Heat Shock Protein 90
HTN: Hypertension
ImmuCellAI: Immune Cell Abundance Identifier
iTDDS: Innovative Drug delivery system
KEGG: Kyoto Encyclopedia of Genes and Genomes
L.E: Ligand efficiency
LogP: Partition coefficient
LUAD: Lung Adenocarcinoma
MAOB: Monoamine oxidase B
MD: Molecular dynamics
MM: Molecular Mechanics
MOE: Molecular Operating Environment
Mwt: Molecular weight
MyoV, VC,5C: Myosin V, VC, 5 C
NAMD: Nanoscale Molecular Dynamics
NCBI: National Center for Biotechnology Information
NCDs: Non-communicable diseases
NK: Natural killer cells
NR: Nuclear receptors (NR) ligands
NSCLC: Non-Small Cell Lung Cancer
NT: Nanotechnology
o/w: Octanol-water
PAH: Pulmonary Arterial Hypertension
PDB: Protein Data Bank
PEG: Polyethylene glycol
PLGA: Polylactic-coglycolic acid
PPAR-gamma/PPARG: Peroxisome Proliferator-Activated Receptor Gamma
PPARA: Peroxisome Proliferator-Activated Receptor Alpha
PPI: Protein-Protein Interaction
QM: Quantum Mechanics
QSAR: Quantitative Structure-Property Relationships
RMSD: Root-Mean Square Deviations
SCLC: Small Cell Lung Cancer
SEA: Similarity Ensemble Approach
SIB: Swiss Institute of Bioinformatics
SMILES: Simplified Molecular Input line entry specification
TCGA: The Cancer Genome Atlas
TDD: Targeted Drug Delivery
TDDS: Targeted Drug delivery system
TISIDB: The integrated repository portal for tumor-immune system interactions
TPSA: Topological polar surface area
TZDs: Thiazolidinediones

## References

[1] Hamdy, N. M., Eskander, G. & Basalious, E. B. Insights on the dynamic innovative tumor Targeted-Nanoparticles-Based drug delivery systems activation techniques. *Int. J. Nanomed.*, 17, 6131–6155 (2022). 10.2147/IJN.S386037 PMC9741821 36514378

[2] Hamdy, N. M., Boseila, A. A., Ramadan, A. & Basalious, E. B. Iron Oxide Nanoparticles-Plant Insignia Synthesis with Favorable Biomedical Activities and Less Toxicity, in the Era of the-Green: A Systematic Review. LID – 10.3390/pharmaceutics14040844 [doi] LID – 844, Pharmaceutics, 14 844. (2022). 10.3390/pharmaceutics14040844 PMC9026296 35456678

[3] Rostamabadi, H., Falsafi, S. R. & Jafari, S. M. Nanoencapsulation of carotenoids within lipid-based nanocarriers. *J. Controlled Release: Official J. Controlled Release Soc.* **298**, 38–67 (2019). 10.1016/j.jconrel.2019.02.005 30738975

[4] Sánchez-López, E., Gómara, M. J. & Haro, I. Nanotechnology-Based platforms for vaginal delivery of peptide microbicides. *Curr. Med. Chem.* **28**, 4356–4379 (2021). 33297908 10.2174/0929867328666201209095753

[5] Herrmann, I. K., Wood, M. J. A. & Fuhrmann, G. Extracellular vesicles as a next-generation drug delivery platform. *Nat. Nanotechnol.* **16**, 748–759 (2021). 34211166 10.1038/s41565-021-00931-2

[6] Chen, G. et al. LncRNADisease: a database for long-non-coding RNA-associated diseases. *Nucleic Acids Res.* **41**, D983–986 (2013). 23175614 10.1093/nar/gks1099 PMC3531173

[7] Kanehisa, M., Sato, Y., Kawashima, M., Furumichi, M. & Tanabe, M. KEGG as a reference resource for gene and protein annotation. *Nucleic Acids Res.* **44**, D457–462 (2016). 26476454 10.1093/nar/gkv1070 PMC4702792

[8] Tsubouchi, Y. et al. Inhibition of human lung cancer cell growth by the peroxisome proliferator-activated receptor-gamma agonists through induction of apoptosis, *Biochemical and Biophysical Research Communications* **270** 400–405. (2000). 10.1006/bbrc.2000.2436 10753637

[9] Chang, T. H. & Szabo, E. Induction of differentiation and apoptosis by ligands of peroxisome proliferator-activated receptor gamma in non-small cell lung cancer. *Cancer Res.* **60**, 1129–1138 (2000). 10706135

[10] Wick, M. et al. Peroxisome proliferator-activated receptor-gamma is a target of nonsteroidal anti-inflammatory drugs mediating cyclooxygenase-independent Inhibition of lung cancer cell growth. *Mol. Pharmacol.* **62**, 1207–1214 (2002). 12391285 10.1124/mol.62.5.1207

[11] Keshamouni, V. G. et al. Peroxisome proliferator-activated receptor-gamma activation inhibits tumor progression in non-small-cell lung cancer. *Oncogene* **23**, 100–108 (2004). 14712215 10.1038/sj.onc.1206885

[12] Keshamouni, V. G. et al. PPAR-gamma activation inhibits angiogenesis by blocking ELR + CXC chemokine production in non-small cell lung cancer. *Neoplasia (New York N Y)*. **7**, 294–301 (2005). 15799829 10.1593/neo.04601 PMC1501135

[13] Akaike, M. et al. The hinge-helix 1 region of peroxisome proliferator-activated receptor gamma1 (PPARgamma1) mediates interaction with extracellular signal-regulated kinase 5 and PPARgamma1 transcriptional activation: involvement in flow-induced PPARgamma activation in endothelial cells. *Mol. Cell. Biol.* **24**, 8691–8704 (2004). 15367687 10.1128/MCB.24.19.8691-8704.2004 PMC516745

[14] Winn, R. A. et al. Antitumorigenic effect of Wnt 7a and Fzd 9 in non-small cell lung cancer cells is mediated through ERK-5-dependent activation of peroxisome proliferator-activated receptor gamma. *J. Biol. Chem.* **281**, 26943–26950 (2006). 16835228 10.1074/jbc.M604145200

[15] ÖztÜrk, N. A. O., Kara, A. A. O. & Vural, A. O. İ, Formulation and In Vitro Evaluation of Telmisartan Nanoparticles Prepared by Emulsion-Solvent Evaporation Technique, (2020). 10.4274/tjps.galenos.2019.76402 PMC7650737 33177929

[16] Mostafa, A. M., Hamdy, N. M., Abdel-Rahman, S. Z. & El-Mesallamy, H. O. Effect of vildagliptin and Pravastatin combination on cholesterol efflux in adipocytes. *IUBMB Life*. **68**, 535–543 (2016). 27251372 10.1002/iub.1510

[17] Mostafa, A. M., Hamdy, N. M., El-Mesallamy, H. O. & Abdel-Rahman, S. Z. Glucagon-like peptide 1 (GLP-1)-based therapy upregulates LXR-ABCA1/ABCG1 cascade in adipocytes. *Biochem. Biophys. Res. Commun.* **468**, 900–905 (2015). 26603933 10.1016/j.bbrc.2015.11.054

[18] Anis, A., Mostafa, A., Kerema, M. S., Hamdy, N. M. & Sultan, A. S. In Silico and cheminformatics prediction with experimental validation of an adipogenesis cocktail, Sorafenib with Rosiglitazone for HCC dedifferentiation. *J. Genetic Eng. Biotechnol.* **22** (4), 100429 (2024). 10.1016/j.jgeb.2024.100429 PMC11600669 39674644

[19] Sasaki, H. et al. Decreased perioxisome proliferator-activated receptor gamma gene expression was correlated with poor prognosis in patients with lung cancer. *Lung cancer (Amsterdam Netherlands)*. **36**, 71–76 (2002). 11891036 10.1016/s0169-5002(01)00449-4

[20] Ciaramella, V. et al. Activity and molecular targets of Pioglitazone via Blockade of proliferation, invasiveness and bioenergetics in human NSCLC. *J. Exp. Clin. Cancer Res.* **38**, 178 (2019). 31027492 10.1186/s13046-019-1176-1 PMC6485164

[21] Stark, C., Breitkreutz, B. J., Reguly, T., Boucher, L. & Breitkreutz, A. Tyers, biogrid: a general repository for interaction datasets. *Nucleic Acids Res.* **34**, D535–539 (2006). 16381927 10.1093/nar/gkj109 PMC1347471

[22] Ru, B. et al. TISIDB: an integrated repository portal for tumor–immune system interactions. *Bioinformatics* **35**, 4200–4202 (2019). 30903160 10.1093/bioinformatics/btz210

[23] Ayza, M. A., Zewdie, K. A., Tesfaye, B. A., Gebrekirstos, S. T. & Berhe, D. F. Anti-Diabetic effect of Telmisartan through its partial PPARγ-Agonistic activity. *Diabetes Metabolic Syndrome Obesity: Targets Therapy*. **13**, 3627–3635 (2020). 33116714 10.2147/DMSO.S265399 PMC7567533

[24] Amano, Y. et al. Structural basis for telmisartan-mediated partial activation of PPAR gamma. *Hypertens. Research: Official J. Japanese Soc. Hypertens.* **35**, 715–719 (2012). 10.1038/hr.2012.17 22357520

[25] Maejima, Y. et al. Telmisartan, a unique ARB, improves left ventricular remodeling of infarcted heart by activating PPAR gamma. *Lab. Invest.* **91**, 932–944 (2011). 21403641 10.1038/labinvest.2011.45

[26] Rasheduzzaman, M., Moon, J. H., Lee, J. H., Nazim, U. M. & Park, S. Y. Telmisartan generates ROS-dependent upregulation of death receptor 5 to sensitize TRAIL in lung cancer via Inhibition of autophagy flux. *Int. J. Biochem. Cell Biol.* **102**, 20–30 (2018). 29929000 10.1016/j.biocel.2018.06.006

[27] Surapaneni, S. K. et al. Telmisartan facilitates the anticancer effects of CARP-1 functional mimetic and Sorafenib in rociletinib resistant Non-small cell lung Cancer. *Anticancer Res.* **41**, 4215–4228 (2021). 34475041 10.21873/anticanres.15226 PMC8691118

[28] Zhang, S. & Wang, Y. Telmisartan inhibits NSCLC A549 cell proliferation and migration by regulating the PI3K/AKT signaling pathway. *Oncol. Lett.* **15**, 5859–5864 (2018). 29552215 10.3892/ol.2018.8002 PMC5840679

[29] Jafari, D. et al. Designer exosomes: A new platform for biotechnology therapeutics. *BioDrugs* **34**, 567–586 (2020). 32754790 10.1007/s40259-020-00434-x PMC7402079

[30] Wang, Z., Wang, Q., Qin, F. & Chen, J. Exosomes: a promising avenue for cancer diagnosis beyond treatment. *Front. Cell. Dev. Biology*. **12**, 1344705 (2024). 10.3389/fcell.2024.1344705 PMC10900531 38419843

[31] Kar, R. et al. Exosome-Based smart drug delivery tool for Cancer theranostics. *ACS Biomaterials Sci. Eng.* **9**, 577–594 (2023). 10.1021/acsbiomaterials.2c01329 PMC9930096 36621949

[32] Zhang, M. et al. Engineered exosomes from different sources for cancer-targeted therapy. *Signal. Transduct. Target. Therapy*. **8**, 124 (2023). 10.1038/s41392-023-01382-y PMC10017761 36922504

[33] Keerthikumar, S. et al. ExoCarta: A Web-Based compendium of Exosomal cargo. *J. Mol. Biol.* **428**, 688–692 (2016). 26434508 10.1016/j.jmb.2015.09.019 PMC4783248

[34] Lv, Y. et al. Nonmuscle myosin heavy chain IIA-Mediated exosome release via regulation of the Rho-Associated kinase 1/myosin light chains/Actin pathway. *Front. Pharmacol.* **11**, 598592 (2020). 33363470 10.3389/fphar.2020.598592 PMC7753194

[35] Bhattacharya, K. et al. Multi-Epitope Vaccine Design against Monkeypox Virus via Reverse Vaccinology Method Exploiting Immunoinformatic and Bioinformatic Approaches, 10 2010. (2022). 10.3390/vaccines10122010 PMC9786588 36560421

[36] Karlsson M, Zhang C, Méar L, et al. A single-cell type transcriptomics map of human tissues.Sci Adv.**7** (31), eabh2169 (2021). 10.1126/sciadv.abh2169 10.1126/sciadv.abh2169 PMC8318366 34321199

[37] Chandrashekar, D. S. et al. UALCAN: an update to the integrated cancer data analysis platform. *Neoplasia (New York N Y)*. **25**, 18–27 (2022). 35078134 10.1016/j.neo.2022.01.001 PMC8788199

[38] Sobin, L. et al. Histologic and Quality Assessment of Genotype-Tissue Expression (GTEx) Research Samples: A Large Postmortem Tissue Collection, Archives of pathology & laboratory medicine, (2024). 10.5858/arpa.2023-0467-OA 38797720

[39] RockweilerN.B. et al. The origins and functional effects of postzygotic mutations throughout the human life span. *Sci. (New York N Y)*. **380**, eabn7113 (2023). 10.1126/science.abn7113 PMC11246725 37053313

[40] Tang, Z. et al. GEPIA: a web server for cancer and normal gene expression profiling and interactive analyses. *Nucleic Acids Res.* **45**, W98–W102 (2017). 28407145 10.1093/nar/gkx247 PMC5570223

[41] Miao, Y. R. et al. ImmuCellAI: A unique method for comprehensive T-Cell subsets abundance prediction and its application in Cancer immunotherapy. *Adv. Sci. (Weinh)*. **7**, 1902880 (2020). 32274301 10.1002/advs.201902880 PMC7141005

[42] Li, T. et al. TIMER: A web server for comprehensive analysis of Tumor-Infiltrating immune cells. *Cancer Res.* **77**, e108–e110 (2017). 29092952 10.1158/0008-5472.CAN-17-0307 PMC6042652

[43] Warde-Farley, D. et al. The genemania prediction server: biological network integration for gene prioritization and predicting gene function. *Nucleic Acids Res.* **38**, W214–W220 (2010). 20576703 10.1093/nar/gkq537 PMC2896186

[44] Kouranov, A. et al. The RCSB PDB information portal for structural genomics. *Nucleic Acids Res.* **34**, D302–D305 (2006). 16381872 10.1093/nar/gkj120 PMC1347482

[45] Innis, C. A. siteFiNDER|3D: a web-based tool for predicting the location of functional sites in proteins. *Nucleic Acids Res.* **35**, W489–W494 (2007). 17553829 10.1093/nar/gkm422 PMC1933183

[46] Ragab, M. A. et al. Structure-based design and synthesis of conformationally constrained derivatives of methyl-piperidinopyrazole (MPP) with Estrogen receptor (ER) antagonist activity. *Bioorg. Chem.* **119**, 105554 (2022). 34923243 10.1016/j.bioorg.2021.105554

[47] Cramer, C. J. (2006).

[48] Wang, J., Wolf, R. M., Caldwell, J. W., Kollman, P. A. & Case, D. A. Development and testing of a general AMBER force field. *J. Comput. Chem.* **25**, 1157–1174 (2004). 15116359 10.1002/jcc.20035

[49] Anwar, M. M., Albanese, C., Hamdy, N. M. & Sultan, A. S. Rise of the natural red pigment ‘prodigiosin’ as an Immunomodulator in cancer. *Cancer Cell Int.* **22**, 419 (2022). 36577970 10.1186/s12935-022-02815-4 PMC9798661

[50] Khodair, A. I. et al. Camptothecin structure simplification elaborated new imidazo[2,1-b]quinazoline derivative as a human topoisomerase I inhibitor with efficacy against bone cancer cells and colon adenocarcinoma. *Eur. J. Med. Chem.* **265**, 116049 (2024). 38185054 10.1016/j.ejmech.2023.116049

[51] Chiang, Y. F. et al. Hinokitiol inhibits breast Cancer cells in vitro Stemness-Progression and Self-Renewal with apoptosis and autophagy modulation via the CD44/Nanog/SOX2/Oct4 pathway. *Int. J. Mol. Sci.*, **25**(7), 3904 (2024). 10.3390/ijms25073904 PMC11011552 38612715

[52] Atta, H. et al. New trends in synthetic drugs and natural products targeting 20S proteasomes in cancers. *Bioorg. Chem.* **133**, 106427 (2023). 36841046 10.1016/j.bioorg.2023.106427

[53] El Mesallamy, H. O., Rashed, W. M., Hamdy, N. M. & Hamdy, N. High-dose methotrexate in Egyptian pediatric acute lymphoblastic leukemia: the impact of ABCG2 C421A genetic polymorphism on plasma levels, what is next? *J. Cancer Res. Clin. Oncol.* **140**, 1359–1365 (2014). 24718721 10.1007/s00432-014-1670-y PMC11823488

[54] Zhang, J., Tang, M. & Shang, J. PPARγ modulators in lung cancer: molecular mechanisms, clinical prospects, and challenges. *Biomolecules* **14** 190. (2024). 10.3390/biom14020190 PMC10886696 38397426

[55] Daniil, S. et al. Differential signaling pathways in medulloblastoma: Nano-biomedicine targeting Non-coding epigenetics to improve current and future therapeutics. *Curr. Pharm. Design*. **30**, 31–47 (2024). 10.2174/0113816128277350231219062154 38151840

[56] Rizk, N. I. et al. Revealing the role of serum Exosomal novel long non-coding RNA NAMPT-AS as a promising diagnostic/prognostic biomarker in colorectal cancer patients. *Life Sci.* **352**, 122850 (2024). 38901687 10.1016/j.lfs.2024.122850

[57] Azimi, F. et al. Tumor-infiltrating lymphocyte grade is an independent predictor of Sentinel lymph node status and survival in patients with cutaneous melanoma. *J. Clin. Oncol.* **30**, 2678–2683 (2012). 22711850 10.1200/JCO.2011.37.8539

[58] Kaur, S., Nag, A., Gangenahalli, G. & Sharma, K. Peroxisome proliferator activated receptor gamma sensitizes Non-small cell lung carcinoma to gamma irradiation induced apoptosis. *Front. Genet.*, **10**, 554 (2019). 10.3389/fgene.2019.00554 PMC6585470 31263479

[59] Hirohara, M., Saito, Y., Koda, Y., Sato, K. & Sakakibara, Y. Convolutional neural network based on SMILES representation of compounds for detecting chemical motif. *BMC Bioinform.* **19**, 526 (2018). 10.1186/s12859-018-2523-5 PMC6311897 30598075

[60] Xiong, G. et al. ADMETlab 2.0: an integrated online platform for accurate and comprehensive predictions of ADMET properties. *Nucleic Acids Res.* **49**, W5–W14 (2021). 33893803 10.1093/nar/gkab255 PMC8262709

[61] Jiang, D. et al. ADMET evaluation in drug discovery. 20. Prediction of breast cancer resistance protein Inhibition through machine learning. *J. Cheminform.* **12**, 16 (2020). 33430990 10.1186/s13321-020-00421-y PMC7059329

[62] Kharkar, P. S. Drugs acting on central nervous system (CNS) targets as leads for non-CNS targets. *F1000Research* **3**, 40 (2014). 24715979 10.12688/f1000research.3-40.v1 PMC3961999

[63] Hitchcock, S. A. & Pennington, L. D. Structure-brain exposure relationships. *J. Med. Chem.* **49**, 7559–7583 (2006). 17181137 10.1021/jm060642i

[64] Julie, N. L., Julie, I. M., Kende, A. I. & Wilson, G. L. Mitochondrial dysfunction and delayed hepatotoxicity: another lesson from troglitazone. *Diabetologia* **51**, 2108–2116 (2008). 18726085 10.1007/s00125-008-1133-6

[65] Floyd, J. S., Barbehenn, E., Lurie, P. & Wolfe, S. M. Case series of liver failure associated with Rosiglitazone and Pioglitazone. *Pharmacoepidemiol. Drug Saf.* **18**, 1238–1243 (2009). 19623674 10.1002/pds.1804

[66] May, L. D., Lefkowitch, J. H., Kram, M. T. & Rubin, D. E. Mixed hepatocellular-cholestatic liver injury after Pioglitazone therapy. *Ann. Intern. Med.* **136**, 449–452 (2002). 11900497 10.7326/0003-4819-136-6-200203190-00008

[67] Daina, A., Michielin, O. & Zoete, V. SwissTargetPrediction: updated data and new features for efficient prediction of protein targets of small molecules. *Nucleic Acids Res.* **47**, W357–W364 (2019). 31106366 10.1093/nar/gkz382 PMC6602486

[68] Daina, A. & Zoete, V. Testing the predictive power of reverse screening to infer drug targets, with the help of machine learning. *Commun. Chem.* **7**, 105 (2024). 38724725 10.1038/s42004-024-01179-2 PMC11082207

[69] Šilhavý, J. et al. Acute toxic effects of Telmisartan in spontaneously hypertensive rats fed a high Fructose diet. *Physiol. Res.* **67**, 851–856 (2018). 30204469 10.33549/physiolres.933951

[70] Irwin, J. J. et al. ZINC20—A free Ultralarge-Scale chemical database for ligand discovery. *J. Chem. Inf. Model.* **60**, 6065–6073 (2020). 33118813 10.1021/acs.jcim.0c00675 PMC8284596

[71] Irwin, J. J. & Shoichet, B. K. ZINC–a free database of commercially available compounds for virtual screening. *J. Chem. Inf. Model.* **45**, 177–182 (2005). 15667143 10.1021/ci049714 PMC1360656

[72] Petitpas, I. et al. Structural basis of albumin-thyroxine interactions and Familial dysalbuminemic hyperthyroxinemia. *Proc. Natl. Acad. Sci. U.S.A.* **100**, 6440–6445 (2003). 12743361 10.1073/pnas.1137188100 PMC164465

[73] Desiere, F. et al. The PeptideAtlas project. *Nucleic Acids Res.* **34**, D655–D658 (2006). 16381952 10.1093/nar/gkj040 PMC1347403

[74] Nascimento, A. F. Z. et al. Structural insights into functional overlapping and differentiation among myosin V motors. *J. Biol. Chem.* **288**, 34131–34145 (2013). 24097982 10.1074/jbc.M113.507202 PMC3837155

[75] Hernández-Rodríguez, M., Correa-Basurto, J., Gutiérrez, A., Vitorica, J. & Rosales-Hernández, M. C. Asp32 and Asp228 determine the selective Inhibition of BACE1 as shown by Docking and molecular dynamics simulations. *Eur. J. Med. Chem.* **124**, 1142–1154 (2016). 27639619 10.1016/j.ejmech.2016.08.028

[76] Fang, Y. et al. Synthesis, biological evaluation, and molecular dynamics (MD) simulation studies of three novel F-18 labeled and focal adhesion kinase (FAK) targeted 5-bromo pyrimidines as radiotracers for tumor. *Eur. J. Med. Chem.* **127**, 493–508 (2017). 28109944 10.1016/j.ejmech.2017.01.015

[77] Ivanov, A. A. et al. Molecular modeling and molecular dynamics simulation of the human A2B adenosine receptor. The study of the possible binding modes of the A2B receptor antagonists. *J. Med. Chem.* **48**, 6813–6820 (2005). 16250640 10.1021/jm049418o

[78] Chen, Y. C. et al. Effect of vitamin D supplementation on primary dysmenorrhea: A systematic review and Meta-Analysis of randomized clinical trials. *Nutrients* **15** 2830. (2023). 10.3390/nu15132830 PMC10343446 37447156

